# Activation of Unfolded Protein Response Pathway in Malignancies: Interplay with Extracellular Matrix and Targeting Perspectives

**DOI:** 10.3390/cancers17121972

**Published:** 2025-06-13

**Authors:** Eleftherios N. Athanasopoulos, Angeliki Natsiou, Maria Kyriazopoulou, Dimitra Manou, Achilleas D. Theocharis, Vassiliki T. Labropoulou

**Affiliations:** 1 Biochemistry, Biochemical Analysis and Matrix Pathobiology Research Group, Laboratory of Biochemistry, Department of Chemistry, University of Patras, 26504 Patras, Greece; up1064177@ac.upatras.gr (E.N.A.); up1068886@ac.upatras.gr (A.N.); up1073746@ac.upatras.gr (M.K.); manou.d@ac.upatras.gr (D.M.); 2 Hematology Division, Department of Internal Medicine, University Hospital of Patras, University of Patras, 26504 Rion, Greece

**Keywords:** unfolded protein response, endoplasmic reticulum stress, extracellular matrix, signaling, multiple myeloma, glioblastoma

## Abstract

Malignant cells exhibit elevated rates of protein synthesis and secretion to facilitate tumor growth, proliferation, and tumorigenesis. Upon malignant transformation, the endoplasmic reticulum (ER) experiences stress due to the accumulation of unfolded or misfolded proteins in the ER lumen, lack of nutrient availability and overall hostile tumor microenvironment conditions. The demand for regulated protein turnover and proteostasis reinstatement results in the activation of the unfolded protein response (UPR) pathway for cellular adaptation and survival. The UPR machinery utilizes the BiP chaperone and three ER-bound sensors, PERK, IRE1, and ATF6, to substantiate signal transduction and orchestrate gene expression associated with protein folding, degradation and recycling, inflammation, autophagy, and programmed cell death. The pleiotropic function of UPR emerges as a central mediator for tumor progression, especially in multiple myeloma and glioblastoma pathologies. Numerous studies have recently pointed out that communication of the extracellular matrix (ECM) with surrounding tumor cells dictates in part UPR activity and vice versa. In the context of this dynamic interplay, ER stress and UPR mechanisms have been proposed as potential targets to elicit novel and effective therapeutic approaches in clinical trials.

## Simple Summary

This review focuses on the roles of the UPR pathway in tumor progression and cell fate determination. Recent insights in UPR signaling have highlighted its diverse function in malignant cells, emphasizing its significance in potential therapeutic approaches. Herein, we also provide increasing evidence that suggests direct and/or indirect synergy between UPR and ECM towards oncogenic signaling, establishing the comprehensive effects of UPR in tumor microenvironment alterations and tumor cell homeostasis. Our aim is to pinpoint the signaling networks in which UPR participates, together with inflammatory responses, apoptosis regulation, and proteostasis mechanisms in malignant cells. Thus, we attempt to gather well-documented and novel knowledge to improve our understanding of the UPR pathway and its implication in tumorigenic potential and aggressiveness of difficult-to-treat tumors, emphasizing multiple myeloma and glioblastoma.

## 1. Endoplasmic Reticulum Stress

The endoplasmic reticulum (ER) is a complex organelle that orchestrates a wide range of cellular functions, including global protein synthesis, modification and trafficking, lipid metabolism, and maintenance of Ca^2+^ homeostasis. The dynamic nature and architecture of the ER, composed of tubules, sheets, and a nuclear envelope, allow it to exhibit its diverse roles as an extensive regulator of cell physiology [1,2]. As the largest organelle of most eukaryotic cells, it is categorized in the rough (RER) and smooth ER (SER), with the former being in close proximity with ribosomes, thus participating in protein synthesis, and the latter being involved mainly in lipid metabolism [3,4,5]. Proteins destined to be transported to the Golgi apparatus, lysosomes, plasma membrane, and extracellular matrix (ECM), as well as the ER, are required to initially enter through the RER. The ER then serves as a quality control center where proteins are being modified and undergo thorough evaluation at the level of their 3D structure prior to reaching their destination. On the other hand, cytosolic, nuclear, and mitochondrial proteins are synthesized on free ribosomes and depend on ER function for their potential post-translational modifications. Notably, extracellular proteins are transported in the ECM microenvironment by being enclosed in ER membrane portions, which are fused with the plasma membrane in order to be secreted [6,7,8,9].

Proteins present in the ER display a plethora of functions, acting as signaling molecules and scaffolds for ER membrane structure, and among others, luminal ER proteins regulate the folding of newly synthesized polypeptides. To ameliorate protein assembly, transport, and function, specific reactions and interactions take place within the ER, such as N-glycosylation, addition of lipid groups, and disulfide bonds [10,11,12,13]. This process is further assisted by molecular chaperones that catalyze the 3D conformation acquisition of synthesized proteins, with binding immunoglobulin protein (BiP, also known as GRP78), a member of the heat shock protein HSP70 family, being the most prominent. BiP innately displays high affinity for unfolded peptides and proteins, therefore facilitating their shape rearrangement, evoked by the hydrolysis of ATP by the ATPase domain, through direct binding. Proteins dissociate from BiP once they acquire the correct 3D structure, rendering BiP a central mediator of global protein folding [14,15,16,17,18]. It has been proposed that tethering of multiple BiP molecules to unfolded proteins optimizes folding in silico, pinpointing a possible rationale for BiP clustering [19]. N-glycosylation also contributes to additional protection from protein aggregation in the ER due to glycans’ size and hydrophilic properties, while simultaneously providing signals for protein secretion [20].

Under physiological conditions, the mechanisms that govern peptide folding are prone to errors, leading to unfolded or misfolded proteins. It is well established that distinct mechanisms within the ER counteract this phenomenon by utilizing chaperones for evaluating the nature of the protein and whether hydrophobic regions are exposed improperly [21,22]. Of importance is the calnexin cycle, in which calnexin and calreticulin chaperones cooperate with glucosyltransferase enzymes to scan proteins and determine their terminal destination [22,23]. Irreversibly unfolded or misfolded proteins are targeted by ubiquitin ligase complexes and translocated back to the cytosol for degradation in the proteasome. Overall, the mechanism that regulates these events is characterized as ER-associated degradation (ERAD) and gets substantiated by the successive steps of protein recognition, retro-translocation, ubiquitination, and degradation [24,25]. Given that protein folding errors manifest themselves ubiquitously, most proteins implicated in ERAD appear to be constitutively expressed and present in their respective ER loci. This quality control that occurs in the ER is not limited to each individual protein but extends to the level of total unfolded protein load estimation and concomitant monitoring of the folding capacity of the ER in order to adapt to cellular demands [26].

Acute or chronic stimuli emerging from physiological or pathological conditions can lead to a state known as ER stress. Considering that the ER’s main purpose is to regulate protein status, Ca^2+^ levels, and lipid metabolism, a disturbance in at least one of those machineries perturbs the integrity and function of the organelle [27]. A study suggests a novel mechanism by which ER stress stabilizes ubiquitin ligases and their interaction with their substrates in order to enhance ERAD activity and adjust cell functionality [28]. Nevertheless, the hallmark of ER stress induction is the accumulation of unfolded or misfolded proteins in the ER lumen that exceeds ERAD and the calnexin cycle’s ability to compensate. The central mechanism that mediates and substantiates the adaptation of cells in ER stress is the unfolded protein response (UPR), as described in detail below [27,29,30,31]. The sum of factors that directly or indirectly induce ER stress varies at causality and level of occurrence. It has been demonstrated that abnormal genetic alterations can prevent the encoding proteins from folding and provide resistance from degradation or a tendency of accumulating in the ER. What is more, mutations that diminish ER-related chaperones’ expression disrupt ER homeostasis. It is also well documented that ERAD-associated errors at the level of expression or function of the implicated biomolecules contribute to the induction of ER stress. Low Ca^2+^ levels counteract the proper assembly of newly synthesized proteins as well as the localization and function of ER-located proteins and chaperones, respectively. Abolished Ca^2+^ concentrations simultaneously upset BiP and calnexin’s interaction with unfolded proteins, thus indirectly affecting multiple folding mechanisms [27,32].

The importance of the ER for maintaining a healthy proteome is also highlighted in a context- and tissue-dependent manner. Normal plasma cells and other cell types that display a high demand from the secretory pathway, either constitutively or under stimuli, are in constant need of precise and reliable ER function. This is also apparent in extreme pathologies such as neurodegenerative diseases, tumorigenesis, and cancer progression. In multiple myeloma (MM) clones, having significantly extended ER and being dependent on ER prior to their oncogenic transformation, the overexpression and excessive secretion of antibodies is a major component of ER stress induction [32]. Apart from that, malignant cells have notably high global transcription and translation demands to satisfy their uncontrolled proliferation that subjects the ER to extensive stress. These endogenous alterations in tumors are accompanied by elevated metabolic rates and reactive oxygen species (ROS) generation that altogether remodel intracellular structures and tumor microenvironment (TME), shaping a hostile milieu that infiltrates the ER membrane’s integrity [27]. In cases of NRAS-mutated melanomas, ER stress induction through mitogen-activated protein (MEK) inhibition has known preferential efficiency, apparent cell death, and improved patient prognosis [33]. ER experiences stress under the influence of other factors associated with malignancies, including nutrient deprivation and hypoxia. Nutrient availability appears to affect ER both at inadequate and excessive supplies. Glucose and glutamine are essential metabolic intermediates involved in N-glycosylation, while glucose is the main molecule initiating catabolic reactions for ATP production, necessary for the energy-requiring protein folding process. On the other hand, lack of O_2_ disallows oxygen-dependent isomerases and other enzymes to catalyze folding, disulfide bond formation, and lipid desaturation, creating a predisposition for ER stress under hypoxic conditions [30,34,35].

These intrinsic cell pathologies are also affected dependently or independently by extrinsic factors, among others pharmaceutical drugs and exposure to radiation. MM patients’ treatment incorporates bortezomib (BTZ), a proteasome inhibitor used as the golden standard chemotherapeutic agent for MM malignancies, exploiting proteasome’s central role in protein recycling and proteostasis, therefore inducing lethal ER stress and cell death [36]. Recent findings demonstrate alternative ER stress-induced mechanisms that promote alterations in death receptor 5 (DR5) and distinct apoptotic regulators’ expression, thus mediating MM cells’ death by natural killer (NK) cells in the MM TME [37]. Distinct factors, such as reticulocalbin, implicated in protein trafficking and ER load regulation, display significant oncogenic effects on GBM progression and maintenance of tumorigenicity [38]. Other studies point out that radiation of glioblastoma multiforme (GBM) samples promotes ER stress both in vitro and in vivo, potentiating radiation as a method for targeted apoptosis mediated by ER stress induction [39,40]. Along the same lines, hypoxic radiosensitive GBM cell lines are predisposed to cell death upon chemically induced ER stress [41]. Surprisingly, evidence suggests that radiation-induced ER stress is not a global threat to GBM cell lines, which, on the contrary, display enhanced survival post-radiation, complexing the extent and context in which ER stress acts as an onco-suppressive factor [42]. A common etiology for ER stress induction that correlates with disease formation and progression as well as with physiological and pathological cell malfunction is inflammation. Simultaneously, inflammation can emerge as a consequence of the above and occur as an ER-stress-induced response. Similarly with its role in tumors, inflammation can offset protective mechanisms to promote apoptotic properties, or it can mitigate threatening conditions to alleviate stress. Thus, it partially regulates the dichotomy between lethal or adaptive and defensive cell fate, depending on the context in which cells perform. Besides, response to ER stress is altogether an exceptionally controlled signaling network that tightly regulates the equilibrium of cell death and survival in a manner that resolves excessive or mild ER stress, respectively [43,44,45,46].

It has been shown that secreted signaling molecules and synthetic organic inhibitors are implicated in ER stress induction in different mammalian cell types. Mouse embryonic fibroblasts (MEFs), mouse β pancreatic cells, islets, and MM cells have been proposed as ideal guide cell lines for ER stress studies, considering their hyperactive secretory capacity. Through utilizing these in vitro models, studies have elucidated biomolecules acting as ER stress inducers in a context-dependent manner. High doses of glucose, similarly with exogenous addition of free fatty acids, result in mild ER stress induction, while treatment with interleukin-1β (IL-1β) and interferon-γ (IFN-γ) cytokines initiates inflammatory response and ER stress. Insulin overexpression in β cells and pancreatic islets, which are responsible for insulin production, modification, and secretion, has been shown to lead to controlled ER stress [47].

Tunicamycin (TM), thapsigargin (Tg), brefedin A, dithiothreitol (DTT), and MG132 are widely used as positive ER stress inducers for research purposes. TM, a UDP-N-acetylglucosamine-dolichol phosphate N-acetylglucosamine-1-phosphate transferase (GPT) inhibitor, is a drug that directly inhibits the first step of N-glycosylation and concomitantly inhibits the downstream cascade of protein modification, exhibiting a crucial role in ER stress development. Tg, with a distinct mechanism of action, specifically inhibits the ER Ca^2+^ ATPase SERCA, leading to significantly reduced Ca^2+^ levels. Brefeldin A induces ER stress by interfering with protein transport from the ER to the Golgi apparatus and forcing the abnormal translocation of Golgi-located proteins back to the ER. A more direct outcome results from DTT treatment, which immediately reacts towards sulfhydryl groups in cysteine residues, being a strong reducing agent, and prevents disulfide bridges from forming. Lastly, MG132 acts as a proteasome inhibitor and induces ER stress by accumulating a pool of unfolded and misfolded proteins into the cytosol [47]. TM and Tg compounds have been relatively standardized for in vitro and in vivo studies, given their effectiveness as ER stress inducers. Through utilizing well-established human metabolic cell lines, it has been concluded that both inhibitors are equally effective in vitro, with TM being superior for in vivo models in regard to ER stress induction and simulation of the associated cell state and metabolic profile [48].

## 2. The Unfolded Protein Response Pathway

The essential cell response to ER stress induction is the activation of the UPR mechanism. This highly conserved and versatile signaling pathway is a central regulator of ER homeostasis, protein folding, and proteostasis, acting via three ER-located transmembrane sensors: double-stranded RNA-activated protein kinase (PKR)-like ER kinase (PERK), inositol-requiring enzyme 1 (IRE1) and activating transcription factor 6 (ATF6). All of these signaling proteins cooperate or perform independently to decrease ER-protein load and relieve stress. Following the main principles of signal transduction, after having the unfolded protein status monitored, UPR integrates and delivers information from the ER to the cytosol and finally into cell nuclei [49]. Under circumstances where protein folding demands surpass capacity but stress is mild, the direct aim is UPR activation towards adaptation through gene expression induction, including numerous enzymes and chaperones. UPR also coordinates ER biogenesis simultaneously with the depression of ER client proteins’ flux into the ER and global translation attenuation at multiple levels [49,50]. Irreversible, excessive, and prolonged ER stress leads to pro-apoptotic signaling and cell death, initiated or mediated by the same UPR sensors, in a pathway characterized as terminal UPR. This dual role of UPR key players, depending on ER stress intensity and upstream stimuli, differentiates the distinct downstream cascade of events and eventually cell fate [51].

**Figure 1:**
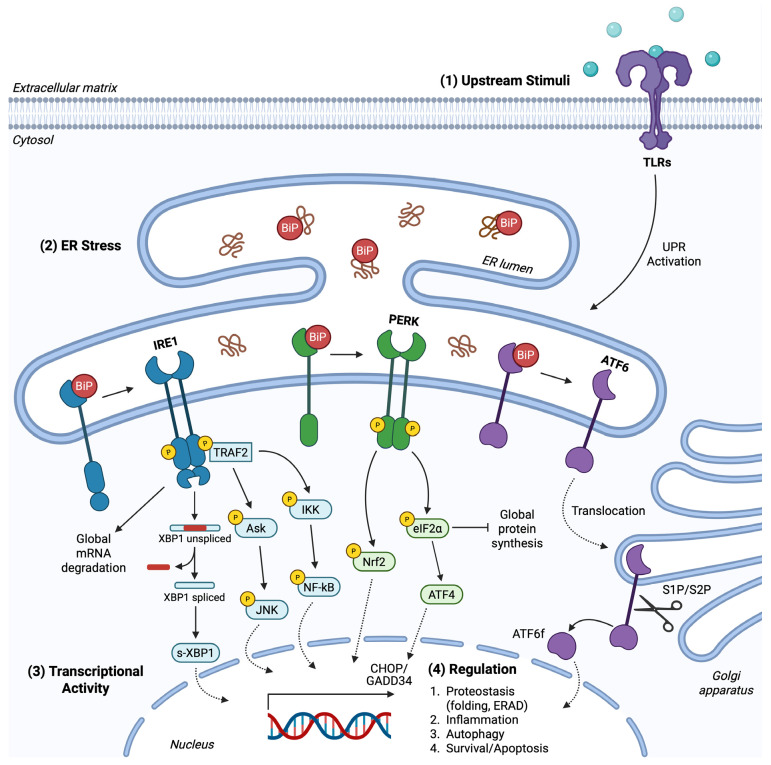
The UPR pathway. (1) Upstream stimuli provoked by transactivation or by ligand–receptor interactions, among others lipopolysaccharide/TLR interaction, provoke UPR signaling. An inflammatory microenvironment, enriched with chemokines and other extracellular inflammatory mediators, excites receptors’ activity towards UPR induction. (2) ER stress, independent of receptor-dependent cascades, promotes the dissociation of BiP from PERK, IRE1, and ATF6 sensors to execute signal transduction. PERK and IRE1 undergo activation upon trans autophosphorylation. PERK inactivates eIF2α through phosphorylation for global translation attenuation and preferential mRNA transcription of ATF4. PERK also phosphorylates and activates Nrf-2 for gene expression induction towards antioxidant response. IRE1 cleaves XBP1 mRNA to produce the active TF s-XBP1, while IRE1 also cleaves mRNAs non-specifically to regulate RIDD. TRAF2 is recognized and attached to phosphorylated IRE1, activating Ask/JNK and IKK/NF-kB pathways. ATF6 is translocated to the Golgi apparatus and cleaved by S1P/S2P proteases, providing the ATF6f product. (3) UPR demonstrates its role via active TFs modulating gene expression. (4) The UPR transcriptional program includes factors involved in folding and protein degradation/synthesis capacity, inflammatory responses, autophagy and cell fate.

The common route for UPR sensing ER stress and undergoing activation involves the BiP chaperone acting as a master regulator of intracellular signal transduction initiation. According to the dominant model regarding all three sensors, BiP directly binds to the luminal domains of PERK, IRE1, and ATF6 under basal conditions, maintaining them in an inactive state. ER stress leads to UPR activation through the dissociation of BiP from UPR sensors (Figure 1) due to the higher affinity that the chaperone displays for unfolded or misfolded proteins accumulated in the ER [50]. This initial step actually highlights BiP as the central ER stress sensor, whereas PERK, IRE1, and ATF6 are indirect and secondary sensors that act as signal transducers [49]. In accordance with this proposition is the fact that BiP overexpression and binding to IRE1 and PERK are associated with their diminished activity, while reduced BiP levels augment UPR signaling in a context-dependent manner [51,52,53,54]. It is also probable that BiP manifests its function as an allosteric regulator of UPR, independently of its chaperone properties that allow it to tether upon exposed hydrophobic protein domains [55,56]. Nevertheless, all three sensors seem to depend on BiP regulation to function in mammalian cells, as confirmed by their overactivation upon mutation-driven inability to bind to BiP, even in the absence of ER stress [55,57,58,59,60].

To decipher the highly sensitive UPR sensor machinery that responds to the slightest cellular alterations, another hypothesis emerged. The second model of the ER stress sensing process arises from the early finding that yeast IRE1 structure includes an MHC-like groove that could potentially sense and bind to extended hydrophobic regions of unfolded proteins [52,61,62]. In addition, distinct but not fully understood components can activate IRE1 in a BiP-independent manner [63]. Thus, it has been proposed that IRE1 (and probably PERK, acquiring similar domains) can interact with unfolded proteins; therefore, this interaction is adequate for sensors’ activation in a direct mechanism of action. Lastly, it has been proposed that the aforementioned models coexist and altogether contribute to UPR activation. The absence of definite literature providing a solid theory consistent for all three UPR sensors independently of BiP and the lack of mechanistical studies demonstrating UPR activation upon direct interaction with unfolded proteins portray the first indirect model, with BiP as the primary sensor, as the most attractive scenario insofar [52,61,63,64].

### 2.1. PERK Branch

PERK kinase belongs to type I transmembrane proteins, consisting as a monomer of an ER-transmembrane domain, an N-terminal ER-luminal domain that interacts with BiP, and a cytosolic domain. The luminal domain, capable of sensing ER stress, is phylogenetically related to IRE1’s respective domain in regard to structure and function [49,65]. Following BiP dissociation, PERK manifests its Ser/Thr kinase activity through its cytosolic portion that undergoes activation upon self-association into dimers, *trans*-autophosphorylation, and subsequent oligomerization [64,66]. Once activated, PERK selectively phosphorylates and inactivates the eukaryotic translation initiation factor 2α (eIF2α), transiently inhibiting global translation and reducing ER-protein influx, thus limiting folding demands and ER load (Figure 1) [49,65]. Mechanistically, phosphorylation of eIF2α at Ser51 exhibits its function indirectly by preventing guanine nucleotide exchange factor eIF2B from recycling total eIF2 to its active GTP-bound form [64]. eIF2α is also a substrate for different kinases activated under ER stress-related conditions. The eIF2αK1, acting as an upstream eIF2α kinase, is involved in regulating stress arising from oxidative or osmotic imbalances, while eIF2αK4 catalyzes eIF2α phosphorylation in response to nutrient starvation and amino acid deprivation [50].

The ubiquitous protein synthesis attenuation, caused by UPR signaling, favors the preferential translation of mRNAs with short open reading frames in their 5′-untranslated regions, expressing, among others, the activating transcription factor 4 (ATF4). This indirect PERK-induced gene expression mediated by ATF4 allows it to positively regulate a large group of UPR target genes associated with amino acid metabolism, mitochondrial protein homeostasis, autophagy, antioxidant response, and protein folding or apoptosis under chronic ER stress, where uncontrolled protein recycling concludes with severe proteotoxicity (Figure 1) [49,65,66]. The main ATF4 target genes include the transcription factor C/EBP homologous protein (CHOP) and growth arrest and DNA damage-inducible 34 (GADD34) (Figure 1). CHOP gene expression leads to further transcription induction involved in cell death and survival. Although PERK and overall UPR signaling is pleiotropic and performs in a context-dependent manner, CHOP expression has been predominantly associated with pro-apoptotic mediators, and specifically CHOP downregulates the anti-apoptotic B-cell leukemia/lymphoma 2 protein (Bcl-2) and induces Bcl-2 interacting mediator of cell death (BIM) and DR5 [64,66,67,68]. Both ATF4 and CHOP enhance GADD34 expression, creating an autoregulatory loop by encoding the GADD34 regulatory subunit of the PP1C phosphatase that dephosphorylates eIF2α, counteracting PERK function. GADD34 forms a negative feedback loop and restricts PERK overactivation; thus, GADD34 serves as a cytoprotective mediator under mild ER stress. Contradictorily, CHOP and GADD34 synergy, or CHOP-induced GADD34 activation, promotes the aforementioned pro-apoptotic pathway leading to ligand-independent, caspase-dependent cell death, therefore utilizing PERK for pro-apoptotic signal transduction [49,64,69,70,71]. Furthermore, ATF4 further oversees PERK signaling by inducing ATF3, and, as is being proposed despite contradictory data, both ATF proteins bind to the GADD34 promoter region. Additionally, in order for ATF4 to successfully promote CHOP expression, not only does the former cooperate with ATF3, but phosphorylation of ATF2 is a prerequisite regulatory reaction for CHOP induction [66,72,73]. This interplay is being further perplexed by recent findings suggesting that GADD34 intriguingly promotes ATF4 and CHOP expression, whereas CHOP can downregulate ATF4 activity, possibly to maintain a tightly regulated network to respond across the spectrum of ER stress intensity [74].

Apart from that, PERK depends on distinct transcription factors (TFs) to mediate ER stress-induced cell signaling. It is worth mentioning that an estimation of around half of PERK target genes’ expression is ATF4 independent, indicating multiple downstream mediators associated with PERK activation. PERK directly activates the nuclear factor erythroid 2-related factor 2 (Nrf-2) through phosphorylation (Figure 1). Nrf-2 is a well-established TF orchestrating redox homeostasis through inducing the encoding of antioxidant enzymes to alleviate ROS accumulation and oxidative stress [49,75]. Under physiological conditions, Nrf-2 remains inactive in the cytosol due to interaction with Keap1 protein. Upon stress, PERK recruits Nrf-2-driven mechanisms to enhance cell survival. Phosphorylated Nrf-2 dissociates from Keap1 in a stable form, accumulates into the nucleus, and binds upon the antioxidant response element (ARE) to induce gene expression, in parallel with ER stress-induced ATF4 function [50]. Mutations at the eIF2α Ser51 residue or UPR activation in PERK-knockout mammalian cells have been proven to be detrimental for cellular adaptation and overall gene expression, which were significantly hindered [76,77]. Simultaneously, it has been reported that perturbations of eIF2α phosphatases’ function, including dysfunctional GADD34 subunits, can either enhance survival capacity or trigger cell death, pinpointing overall the central role of the PERK branch in ER stress response [78,79]. Altogether, the reversible modification of eIF2α phosphorylation and the balanced regulation of its status between a relaxed and a hyperactive state provide a PERK-driven, sensitive response upon both subtle and extreme fluctuations of ER and cellular homeostasis.

### 2.2. IRE1 Branch

IRE1 is the most evolutionarily conserved and well-studied sensor from the three UPR arms. In accordance with PERK, it belongs to the type I transmembrane proteins, and its structure includes a stress-sensitive luminal region at the N-terminal domain, the ER-transmembrane portion, and the cytosolic domain. Although similar, IRE1 significantly differentiates from PERK function, as it possesses simultaneously two enzymatic activities. Firstly, IRE1 acts as a Ser/Thr kinase, catalyzing exclusively its activating *trans*-autophosphorylation reaction, without recognizing different substrates to phosphorylate. Secondly, it exhibits its essential role in part via its endoribonuclease (RNase) activity and the widespread series of events this activity entails [49,50,80]. What is more, IRE1 differs from PERK as it is found expressed in two homologs in mammalian cells, IRE1α and IRE1β. Only the former is expressed in a variety of tissues and participates in the UPR mechanism [81]. As already mentioned, IRE1 activation can be BiP-independent, but the dissociation of the latter is the main signal for the UPR pathway. IRE1/BiP interplay also occurs at the level of expression, as reported by findings that BiP levels elevate due to IRE1 activation in order to restore homeostasis and restrict IRE1 overactivation in a negative feedback loop. Thus, the monomeric IRE1 protein alters conformation upon BiP uncoupling, homodimerizes, concomitantly undergoes autophosphorylation through its kinase activity, and oligomerizes to display its RNase activity under the indirect guide of BiP [82].

The bifunctional effects of its RNase activity are primarily displayed in the nonconventional splicing of the X-box binding protein 1 (XBP1) under ER stress. IRE1 cleaves XBP1 mRNA and, more specifically, excises a 26-nucleotide sequence intron to shift the translational open reading frame and promote the spliced-XBP1 (s-XBP1) translation (Figure 1) [61,65]. The stable generated product then acts as a potent basic leucine zipper (b-ZIP) TF to substantiate an adaptive response. It translocates into the nucleus, forming homodimers and inducing chaperones and enzymes’ gene expression to enhance lipid metabolism, autophagic processes, the ERAD mechanism, protein secretion, modification, and folding overall [50,83,84,85]. This vast IRE1-induced downstream cascade is partly regulated through the ability of the unspliced XBP1 (u-XBP1) unstable product to bind and suppress s-XBP1 function, therefore restraining IRE1-dependent gene expression [65,86]. The u-XBP1 encodes a hydrophobic portion in its protein structure that the s-XBP1 lacks; thus, the latter is not anchored on the ER membrane but is soluble in the cytosol and unbound from u-XBP1, acting as a TF. As mentioned in detail below, ATF6 also utilizes the abolishment of hydrophobic regions with different mechanisms that allow it to manifest its function in the cytosol and nuclei [87].

The secondary machinery that IRE1 initiates to abrogate ER stress independently of XBP1 modification and function is the regulated IRE1-dependent mRNA decay (RIDD). RIDD serves as a robust coordinator to safeguard cellular homeostasis through ubiquitous translation regulation and highly selective mRNA degradation upon IRE1 overexpression or hyperactivation. Among others, mainly mRNAs encoding secretory and ER-located proteins are targeted for degradation by the IRE1 RNase domain, leading to the attenuation of ER influx and load (Figure 1). There have been significant exceptions, though, pointing out that cytosolic, ribosomal, nuclear-resident RNAs, tRNAs, and miRNAs can also undergo RIDD-dependent splicing [80,88]. RIDD-induced miRNA degradation reaffirms findings that highlight the regulatory role of RIDD by cleaving inhibitory miRNAs to promote their target genes’ expression. Furthermore, RIDD constructs a perinuclear cluster of lysosomes that efficiently discharges protein aggregates by inactivating the biogenesis of lysosome-related organelles 1 subunit 1 (Blos1) mRNA [69]. These underline the fact that the RIDD approach not only aims at maintaining proteostasis directly but also provides an adaptive response by reducing folding demands and allowing ER to function properly [50]. To ameliorate the functional aspect of RIDD, IRE1 utilizes specific cleavage sites that RNAs bear, containing a consensus sequence along with a stem-loop structure, and as a consequence, IRE1 recognizes and catalyzes the selective cleavage [89]. This sequence is also present in XBP1, elucidating at least in part the resemblant mechanism by which mRNA hydrolysis occurs at both XBP1-mediated and RIDD pathways. Following the IRE1 endoribonucleolytic reaction, the respective mRNA fragments are further processed by exoribonucleases for complete degradation towards inactivation. In these continuous catabolic reactions, both IRE1α/β isoforms have the capacity to cleave the plethora of mRNAs targeted upon RIDD activation [66,81,88]. It is also notable that the RIDD goal corresponds to eIF2α’s aim for global translation inhibition, but instead of acting at the translation initiation step, it diminishes substrates’ availability and abundance for protein synthesis [51].

An additional special feature that IRE1 possesses is its ability to interact with a variety of factors, thereby regulating a wide range of cellular processes in which these factors participate, regardless of its RNase activity [90]. Bilaterally, proteins, such as chaperones, tethering upon IRE1 can regulate IRE1 signaling. Chaperone HSP90 has been shown to directly bind to IRE1 to prevent proteasome degradation and UPR signaling silencing, whereas chaperone HSP72’s binding enhances IRE1 RNase activity and counteracts the apoptotic pathway [91,92]. On the other hand, IRE1 recruits at its cytosolic C-terminal domain the adaptor protein tumor necrosis factor (TNF) receptor-associated factor 2 (TRAF2), activating the apoptosis-signal-regulating kinase 1 (ASK1) and, in turn, the c-Jun N-terminal kinase (JNK) pathway (Figure 1) [93]. Along the same lines as PERK signaling, IRE1 is capable of initiating both anti- and pro-apoptotic pathways. More precisely, apart from JNK, all mitogen-activated protein kinases (MAPK), extracellular signal-regulated kinases (ERK), p38, and Akt, independently of the mammalian target of rapamycin (mTOR), can undergo activation under IRE1-induced stimuli to coordinate apoptosis in a context-dependent manner [94,95]. Contrarily, upon different stimuli, mTORC1 acts upstream of IRE1 to induce UPR signaling and JNK-dependent gene expression, reduce Akt phosphorylation, and engage in induced cell death [96]. Given the intimate role of inflammation in cell death and survival balance, IRE1 is also involved in inflammatory responses as a means to determine cells’ destiny. IRE1 kinase activity permits the inhibitor of nuclear factor-kB kinase (IKK) to mediate under basal activity the enhancement of NF-kB signaling and regulate apoptosis through JNK, pinpointing simultaneously the crucial role of IRE1 in apoptosis and the importance of its phosphorylation status for cellular homeostasis under ER stress (Figure 1) [97]. This complicated crosstalk is expanded at the initiative for signal transduction, affecting receptors’ function in a ligand-independent manner, as shown by data that IRE1 interacts and transactivates TNF receptor 1 (TNFR1) upon ER stress to transmit apoptotic messages through JNK [98]. IRE1 utilizes JNK as the common executioner that regulates BIM and Bcl-2 expression, as well as the mitochondrial and caspase-dependent apoptotic cascade with Bcl-2-associated X protein (BAX) and Bcl-2 antagonist/killer protein (BAK) triggering cell death via the apoptosome formation. Overall, IRE1, either dependently or independently of XBP1 and RIDD, impacts a wide array of phenomena to compensate for ER and cellular malfunction as a pro-survival response, implicated in proliferation, metabolism, autophagy, and inflammation, or eventually activates terminal UPR, leading to programmed cell death upon extreme ER stress [87].

### 2.3. ATF6 Branch

ATF6 is a type II ER-transmembrane glycoprotein found in oxidized monomer, dimer, and oligomer form that bears no enzymatic activity, in contrast with the other UPR sensors. Two isoforms, ATF6α and ATF6β, have been characterized in mammals, with the former being predominantly implicated in ER stress response and both single-passing through the ER membrane. As already mentioned, ATF6 remains inactive through direct binding of its luminal domain with BiP, while additional intra- and inter-molecular disulfide bridges stabilize its ER-localized structure. The presence of these disulfide bonds renders ATF6 susceptible to the existing reducing conditions that perturb its folding within the ER [60,69,82]. The cytoplasmic domain contains a bZIP motif providing the ability of DNA binding, as well as a transactivation domain, acting as a scaffold for different proteins. Thus, active ATF6 functions as a TF for UPR-induced gene expression. BiP/ATF6 disassembly leads to ATF6 complexes’ dissociation, further enhanced through the reduction of their bridging disulfide bonds, creating a monomer arranged for activation [99]. The imperative for this sequential activation is ATF6 translocation to the Golgi apparatus, ameliorated through two ATF6 Golgi localization signals that BiP disengagement unmasks (Figure 1). Co-chaperones also regulate the ATPase cycle of BiP, and enzymes introduce reducing agents to the ATF6 luminal domain, acting complementary with BiP itself for ATF6 activation. These events are followed by the physical interaction between ATF6 and coat protein complex II (COPII), the sole vesicular protein responsible for packaging and trafficking ATF6 to the Golgi apparatus [58,100,101].

ATF6 arrives at the Golgi in a reduced, monomeric state, where it acts as a substrate successively for Site-1 (S1P) and Site-2 (S2P) Golgi-resident proteases (Figure 1). S1P and S2P proteolytically cleave the luminal and transmembrane ATF6 domains, respectively, in a mechanism known as regulated intermembrane proteolysis (RIP) that describes the process upon which bioactive fragments with TF properties derive from proteolytic hydrolysis [102]. S1P is anchored in the Golgi membrane, being in close proximity with ATF6 and displaying serine protease activity, while S2P metalloprotease catalyzes the reaction producing the active N-terminal ATF6 fragment (ATF6f). Free cytosolic ATF6f is afterwards translocated into the nucleus and induces gene expression associated with chaperone-mediated folding, protein modification, lipid synthesis, ERAD, and autophagic components (Figure 1). Moreover, ATF6f can heterodimerize with distinct TFs, such as the cAMP-response element binding protein (CREB), to broaden the regulation of distinct potential target gene subgroups. An intriguing example concerns the assembly of ATF6f with CREB-hepatocyte (CREBH) in hepatocytes’ nuclei to promote inflammatory proteins’ secretion upon ER stress, to supervise glycolysis/gluconeogenesis equilibrium, lipid metabolism, cell proliferation, and liver’s homeostasis overall through gene expression [99,101,102,103,104].

ATF6 is mainly incorporated in the adaptive UPR to administer a cytoprotective function. Bcl-2 protein ensures cell fidelity and anti-apoptotic role upon ER stress partly due to ATF6-induced expression of the regulator of calcineurin 1 (RCAN1), initiating a cascade of modifications that leads to inactivation of Bcl-2 antagonist of death (BAD) and grants a pro-survival response [81,105]. In regard to anti-apoptotic events, ATF6 activates mTOR signaling for pro-survival cues upon ER stress as a result of its proteolytic degradation in the Golgi apparatus by S1P/S2P proteases and induced transcription [106]. The transcriptional program that ATF6 synchronizes cannot manage protein ER influx directly; therefore, it reforms ER sustainability by inducing ER and Golgi biogenesis and concomitant expansion simultaneously. Although ATF6 does not undergo a reversible regulatory modification in accordance with PERK and IRE1 phosphorylation, ATF6-driven recovery processes are under constant and dynamic adjustments. Interestingly enough, ATF6 protein exists in two isoforms, with ATF6β suppressing ATF6α, providing endogenous mechanisms to overturn ATF6 overactivation [107]. ATF6 irreversible cleavage is also counteracted by the immediate ATF6f degradation in the proteasome, only upon functional TF activity [61,101,108,109]. All the above emphasize the fundamental task of ATF6 as a multitasking mediator of proteostasis and cellular physiology maintenance under utmost and hostile conditions.

### 2.4. UPR Crosstalk

It is apparent that the UPR mechanism not only recruits distinct signaling pathways to substantiate an ER stress-induced response but can also undergo direct activation by multiple phenomena and organize cellular function, acting as a signal transduction initiator, mediator, or executioner. A decisive parameter for this synchronized adaptive endeavor is the crosstalk that interconnects all three UPR branches. PERK, IRE1, and ATF6 share the common principle of utilizing TFs for the regulation of gene expression. What is more, the mechanisms that govern UPR activation elucidate the resemblance among specific UPR components, such as the requirement of discharging hydrophobic domains for uncoupling from the ER membrane in both XBP1 and ATF6 proteins. Along the same lines, all UPR arms utilize an alternative regulatory system for a transient and monitored response, including reversible PERK, eIF2α, and IRE1 phosphorylation and downstream inhibitory networks, ATFα/β opposing function, ATF6f degradation, and negative feedback loops for autoregulatory signal silencing [110].

The similarities between XBP1 and ATF6 TFs led to further investigation, upon which a synergistic and co-dependent relationship emerged. s-XBP1 forms heterodimers with ATF6f for inducing diverse genes’ expression, whereas u-XBP1 complexes with either s-XBP1 or ATF6f promote total structure’s degradation and inactivation [111]. Additionally, ATF6f enhances XBP1 expression that IRE1 cleaves afterwards, while BiP, the UPR nonconventional inhibitor, is also a target gene of ATF4 and ATF6f [112,113]. In parallel, ATF4 can target IRE1 genetic loci and promote its transcription, forming overall a PERK/ATF6 synergy to ameliorate IRE1 signaling [114]. Among others, s-XBP1 positively regulates the DnaJ protein P58IPK homolog, which has been shown to negatively affect PERK activity, forming an overlapping negative feedback loop [83]. The PERK/ATF4 pathway has also proven to be detrimental for ATF6 expression and transport to the Golgi apparatus in a context-dependent manner [115]. Collaterally, both ER stress and ATF6 absence independently can lead to IRE1 upregulation and XBP1 activation as a global response, while IRE1 or JNK kinase activity malfunctions negatively regulate IRE1 levels and pathway, signifying a possible positive autoregulatory control [116]. This ATF6/IRE1 collaboration is a mutual one, upon which ATF6 produces an abundance of XBP1 mRNAs as a prerequisite for IRE1 RNase to be hyperactive. Of importance was the finding that PERK persistent signaling predominantly correlates with ATF4/CHOP-dependent cell death upon inert IRE1/XBP1 function, whereas the opposite is not true. IRE1 can mitigate or completely negate pro-apoptotic pathways in a PERK-inhibited setting. RIDD-induced DR5 mRNA cleavage has a definite role in this IRE1-driven survival programming, but nevertheless these data elicit IRE1 as the potential central UPR sensor that utilizes the overall mechanism for its pathway to function properly [117].

There is evidence suggesting that UPR complements the ubiquitin-proteasome system (UPS) under perturbed proteasome function. The accumulation of non-degraded protein aggregates provokes UPR signaling to alleviate UPS malfunction and restore proteostasis. UPR activity is also essential for UPS/autophagy interconnection through IRE1/JNK/Bcl-2/Beclin-1 activation and global protein recycling [118]. Distinct E3 ligases have been shown to display differential effects on UPR branches through protein targeting and ubiquitination towards ER stress attenuation or excitation. IRE1, CHOP and Bcl-xL proteins have been found to be ubiquitinated to undergo stability and activity regulation. The carboxy-terminus of Hsc70 interacting protein (CHIP) ubiquitinates IRE1 to promote IRE1/TRAF2 interaction and concomitant inflammatory response, whereas hydroxymethyllutaryl reductase degradation protein 1 (HRD1) downregulates CHOP and IRE1 levels, simultaneously with IRE1 phosphorylation and RIDD downregulation, to suppress terminal UPR [119]. In addition, ubiquitin-like protein 5 (UBL5) is susceptible to proteolytic degradation upon PERK signaling, which in turn results in ER stress-induced cell death, highlighting a probable antagonistic role between pro-survival UPS and terminal UPR [120]. Along the same lines, ubiquitin-specific protease 19 (USP19), a member of the deubiquitinases (DUBs) superfamily, is accumulated in the ER in response to ER stress and UPR activation to rescue ERAD substrates, promote protein folding, and act directly as a chaperone [121].

## 3. UPR in Brain and Blood Malignancies

### 3.1. Glioblastoma

#### 3.1.1. Integrated UPR and Tumorigenicity

**Figure 2:**
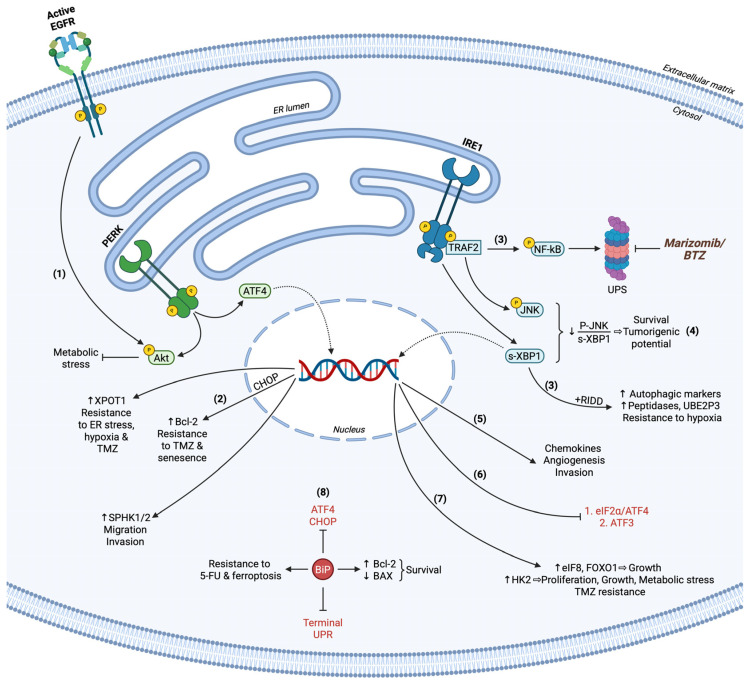
Pro-survival UPR in GBM. (1) PERK activation together with EGFR signaling regulates Akt phosphorylation status to relieve metabolic stress. (2) ATF4-related transcription induces XPOT1 and SPHK1/2 expression, providing pro-survival cues and facilitating migration and invasion, respectively. CHOP upregulates Bcl-2 and cooperates with ATF4, rendering GBM cells resistant to TMZ, ER stress, hypoxia, and senescence. (3) IRE1 synchronizes the transcription rates and secretion of chemokines for angiogenesis and invasion promotion. (4) Active IRE1 restricts terminal UPR by suppressing eIF2α/ATF4 and ATF3 pro-apoptotic functions. (5) Upon persistent XBP1 splicing and attenuated JNK activation through IRE1, GBM cells provoke proliferation and survival capacity. (6) All IRE1-induced mechanisms concerning RIDD, XBP1 modification, and NF-kB activation stimulate autophagy marker expression and autophagic flux, while NF-kB also orchestrates UPS-dependent protein recycling. UPS inhibitors BTZ and marizomib have been utilized in clinical trials for GBM therapy. (7) s-XBP1 gene targets eIF8, FOXO1, and HK2 enhance GBM growth, TMZ resistance, and survival upon hypoxic conditions. (8) BiP acts as a global inhibitor of terminal UPR and ATF4/CHOP effectors, maintaining low BAX/Bcl-2 ratio to hamper pro-apoptotic phenomena. BiP also demonstrates its cytoprotective role against acidic TME by compensating 5-FU chemotherapeutic effects and ferroptosis. Terminal UPR markers are indicated in red.

The importance of an integrated UPR mechanism in tumor progression and survival has been pinpointed in numerous studies concerning GBM pathology [122,123]. Primary glioma cells and xenograft tumors constitutively express all UPR-related chaperones, markers, and transcript targets. Heterogeneous patient samples displayed global UPR hyperactivation linked to excessive secretory capacity and aggressive properties, being further overexpressed in ER stress-induced conditions. U87 cells with different epidermal growth factor receptor (EGFR) expression profiles maintain elevated UPR activity and provide temozolomide (TMZ) resistance upon ER stress. Both GBM grade and characterization into proneural, proliferative, and mesenchymal subgroups identify UPR-associated mediators as markers that correlate with grade IV, mesenchymal, and proliferative GBM, implying poor prognosis and overall survival. U87 cells facilitated pro-survival efforts and chemoresistance, probably due to progressing into a hypermetabolic state through constitutive and persistent UPR signaling [124]. Sphingolipid metabolites and intermediates have been proposed as potential targets for cancer therapy through utilizing novel inhibitory agents [125]. Sphingosine kinase 1/2 (SPHK1/2) enzymes implicated in S1P biosynthesis and transport have been characterized as ATF4 targets in GBM cells. LN229 and U87 cell lines depend on active PERK/eIF2α/ATF4 signaling to induce SPHK1/2 expression, thus inducing GBM migratory and invasive properties (Figure 2). TMZ-resistant GBM cells expand this relationship to promote their aggressive phenotype and enhance the existing TMZ resistance by upregulating SNAI2, N-cadherin, and vimentin EMT markers. These phenotypic characteristics overall provide TMZ chemotherapy evasion and survival in vivo [126]. Of importance was the finding that IRE1 promotes GBM proliferation capacity and resistance to cell death by inducing gene expression. Among others, IRE1 kinase and mostly RNase activity positively affect eIF8 and forkhead box transcription factor 1 (FOXF1) to augment tumor growth and aggressive properties through gene expression (Figure 2). Besides targeting survival-associated genes, IRE1 enzymatic activities correlate with ATF3 downregulation for inhibiting terminal UPR. IRE1-deficient GBM models, in both kinase and RNase activities, render GBM susceptible to ER stress-induced cell death due to the inability of transcription regulation of the above, leading to impaired proliferation and apoptotic phenotype (Figure 2) [127].

Given that GBM tumors undergo aerobic metabolism and function under hypoxic conditions, T98G cells were evaluated for their response to hypoxia induction and resistance to therapy. RNA sequencing and bioinformatics analysis revealed alterations in numerous genes’ expression concerning metabolism and pro-survival events, with IRE1 being significantly upregulated to oversee the above [128]. UPR has also been associated with GBM recurrence in vivo after therapy-induced senescence (TIS) reversion. The Bcl-2 anti-apoptotic marker has been found upregulated in GBM cells post-therapy, signifying transient senescence, maintenance of growth, and cell death resistance due to PERK activation. PERK signal transduction was substantiated by CHOP-induced gene regulation for apoptosis inhibition, whereas loss of PERK in combination with radiation led to unresolved ER stress and persistent senescence, disallowing significant recurrence (Figure 2) [129]. PERK/eIF2α/ATF4 activation is correlated with GBM progression from grade III to grade IV, while rat tumor xenografts exhibit hyperactive PERK in comparison to normal rat brain samples. U87 and U251 cells undergo apoptosis initiation upon TM treatment and PERK suppression, with the combination of both having a significantly enhanced pro-apoptotic result. PERK inactivation was responsible for metabolic stress and cell death, in which Akt activation was hindered in stressed GBM cells. These results were reflected in in vivo experiments, reaffirming the central role of PERK in glioma growth, whereas epidermal growth factor (EGF) exogenous addition partially rescued apoptotic events mediated by PERK loss (Figure 2) [130].

IRE1 is actively implicated in the UPS system by regulating autophagy markers and ubiquitin-specific peptidases and activating enzymes’ expression for orchestrating substrate recognition and degradation. These substrates are highly affected under hypoxic conditions in an IRE-dependent manner to promote GBM tumor growth, supporting relevant data for targeting the UPS system towards GBM therapy [131,132]. C6, U87, and U251 cells recruit s-XBP1 TF activity to organize glycolysis under both normoxic and hypoxic conditions, contributing to aerobic metabolism occurrence for cell viability, growth, proliferation, and resistance. Hexokinase 2 (HK2), a well-established metabolic enzyme required for GBM aggressive properties, was found to be downregulated in XBP1-depleted cells, implying its role as the executioner of XBP1-dependent metabolism shift for ensuring GBM survival upon hypoxia in vitro and in vivo (Figure 2) [133]. Being tightly linked to pro-inflammatory response, IRE1 was also studied for its involvement in immune regulation in GBM TME. Interestingly enough, XBP1 cleavage as well as RIDD were responsible for the expression of the ubiquitin-conjugating E2 enzyme UBE2D3, which entails NF-kB activation. IRE1 initiated a novel axis via NF-kB for chemokine expression and secretion to induce chemotactic attraction of monocytes, macrophages, and neutrophils, thus promoting GBM invasion and angiogenesis (Figure 2) [134]. Novel inhibitors, among others the Z4P IRE1 kinase inhibitor, have emerged as means for GBM sensitization to therapy, such as TMZ treatment to block growth and relapse-free survival, while simultaneously being non-toxic, blood–brain barrier (BBB) permeable, and targeted [135]. LN229 and G55 cells utilize the ATF4 pro-survival function upon hypoxia and ER stress induction, as well as TMZ treatment, through inducing gene expression of exportin-T (XPOT), tryptophanyl-tRNA synthetase 1 (WARS1), and tribbles homolog 3 (TRIB3), providing resistant properties to GBM tumors [136]. Similarly, targeting s-XBP1 in U87 and A172 GBM cells potentiated TMZ efficacy [137].

The pro-survival response that UPR regulates can be counteracted through synchronized terminal UPR function, pinpointing the dichotomy of UPR mechanism in cell death and homeostasis. For instance, nuclear import receptor karyopherin β1 (KPNB1) apoptotic functions impede UPR activity in GBM U87 and U251 cells, whereas KPNB1 depletion initiates terminal UPR signaling to inhibit anti-apoptotic regulators and promote pro-apoptotic pathways via Bcl-2 protein family regulation. The PEKR/eIF2α/ATF4/CHOP pathway was mainly recruited for phorbol-12-myristate-13-acetate-induced protein 1 (NOXA) and p53-upregulated modulator of apoptosis (PUMA) upregulation. Terminal UPR was counterbalanced through autophagy, enhanced poly-ubiquitination, and proteasome-dependent protein recycling for reestablishing proteostasis [138]. GBM specimens tend to overexpress BiP, and it is evident that its levels correlate with GBM growth and proliferation capacity in vitro and in vivo. BiP exhibits cytoprotective roles against TMZ partially by suppressing CHOP expression and function for apoptosis initiation. Furthermore, BiP depletion sensitized GBM samples to treatment with chemotherapeutics, such as 5-fluorouracil (5-FU), highlighting a novel mechanism for targeting specific UPR arms towards GBM therapy (Figure 2) [139]. UPR inhibition prior to ER stress induction in chemotherapy has emerged as an attractive perspective of ongoing research. U87 and LN229 GBM cells have been utilized for blocking BiP and inducing ER stress, highlighting terminal UPR activity in response to stress, significantly enhanced upon BiP inhibition in vitro and in vivo. Although BiP inhibition was inadequate for UPR activation, ER stress and especially synergistic co-treatment led to proliferation and growth inhibition through cell cycle arrest, as well as PERK/eIF2α/ATF4/CHOP and IRE1/XBP1-induced apoptosis, mediated by BAX upregulation and Bcl-2 downregulation (Figure 2) [140]. In thirteen GBM cell lines, BiP was found to be a central mediator of survival, acting in response to acidic TME to decrease ATF4 activity for suppressing ferroptosis (Figure 2) [141].

Even though GBM differentiation can offset the apoptotic mechanisms induced by ER stress, it has been recorded that glioma stem cells (GSCs) are radioresistant due to their stemness capacity regulated in part by UPR activation [142]. The expression pattern of microtubule-associated protein 1A/1B-light chain 3 (LC3), Beclin-1, BiP, GRP94, and CHOP, which were found upregulated upon radiation exposure to safeguard adaptation upon long-term stress, are correlated with poor prognosis of GBM patients. On the contrary, persistent ER stress induced by radiation and 2-deoxy-D-glucose (2-DG) treatment, acting as an ER inducer, significantly reduced cell viability and altered cells’ phenotype in a dose-dependent manner through blocking pro-survival autophagy and excessively enhancing apoptotic UPR [143]. Furthermore, MKC8866, a widespread IRE1 RNase activity inhibitor, has been proven effective for sensitizing GBM cells to irradiation and chemotherapy both in vitro and in vivo. Loss of RNase activity suppressed IRE1 pro-survival properties, especially post-therapy with TM for ER stress induction or TMZ, acting as an onco-suppressor by XBP1 and eIF2α/ATF4 terminal signaling [144]. Patient-derived GBM and immune cells overexpress UPR markers and utilize IRE1a to organize downstream pro-apoptotic and pro-survival mechanisms through JNK1 and XBP1, respectively. More specifically, a correlational relationship was observed, in which patients with a high JNK1/XBP1 expression ratio had better survival outcomes, whereas a low ratio signified resistance to cell death and tumorigenicity, suggesting immunosuppressive TME [145].

#### 3.1.2. Terminal UPR in Glioblastoma

**Figure 3:**
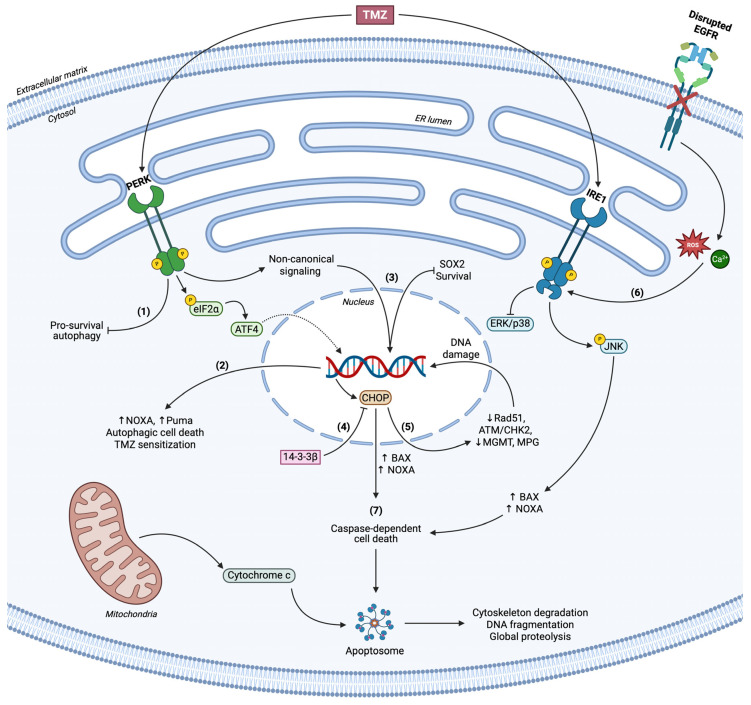
Roles of terminal UPR in GBM. (1) PERK inhibits pro-survival autophagy in a context-dependent manner. (2) PERK/eIF2α/ATF4 axis upregulates NOXA and Puma levels, coordinates autophagic cell death and substantiates TMZ-related apoptosis. (3) Non-canonical PERK signaling modulates SOX2 levels in response to TMZ treatment and results in stemness regulation and loss of viability. (4) CHOP overexpression correlates with the apoptotic phenotype of GBM cells upon ER stress, counteracted through 14-3-3β chaperone. (5) CHOP reduces DNA damage repair enzymes’ levels and activity (Rad51, MGMT, MPG, ATM/CHK2) and accelerates genome instability. (6) Loss of active EGFR signaling concludes to excessive ROS and Ca^2+^ accumulation and concomitant ER stress. IRE1 mediates cell death via JNK activation and simultaneous suppression of ERK/p38 signaling, in parallel with CHOP, to advance BAX and NOXA function. (7) Terminal UPR initiates caspase-dependent cell death.

GBM specimens from patients revealed that ATF4, but not BiP and XBP1, expression was correlated with poor prognosis. Neurosphere formation and viability were significantly attenuated upon Tg treatment, accompanied by caspase-dependent terminal UPR activation of all three branches. Surprisingly, differentiated GBM cells were significantly more resistant to ER stress-induced cell death, whereas UPR-mediated signaling led to apparent GSC apoptosis and necroptosis. PERK was found to be the key player in the initiation of cell death, acting through SOX2 stemness marker’s expression regulation (Figure 3). ER-stress-induced SOX2 downregulation was PERK dependent but eIF2α/ATF4/CHOP independent, implying direct UPR-dependent and UPR-independent mechanisms upon which PERK participates in gene expression, stemness, and viability in GBM [146]. TMZ-treated U87 cells have also exhibited pro-apoptotic PERK/eIF2α/ATF4/CHOP cascade activation to promote NOXA expression and mitochondrial-dependent cell death (Figure 3). ATF4 did not only act as a mediator for TMZ-induced cell death but further enhanced TMZ function for reducing mitochondrial membrane potential [147]. In different settings, EGFR inhibition via gefitinib treatment in H4 and U87 glioma cells led to apparent ROS, free Ca^2+^ intracellular ion generation, and caspase-dependent apoptosis, mediated by global UPR activation and especially IRE1/Ask/JNK upregulation, in parallel with decreased MAPK/Akt signaling (Figure 3). NADPH oxidases 2/4 (NOX2/4) levels were also higher in gefitinib-treated cells. IRE1-depleted cells partially rescued the gefitinib-induced pro-apoptotic pathway, confirming its crucial role in context-dependent cell death. On the contrary, chemically induced ER homeostasis restoration significantly alleviated the H4 response to block terminal UPR function, whereas MAPK/Akt inhibition pointed out their pro-survival involvement in GBM cells upon EGFR inhibition. In U87 cells, UPR activation was accompanied by NOXA overexpression, acting as a link between UPR/mitochondrial-induced apoptosis. The aforementioned findings suggest that EGFR inhibitors elevate intracellular free Ca^2+^ and ROS concentration, leading to NOX2/4 activation and ER stress. Thus, terminal UPR branches, PERK/eIF2α/ATF4/CHOP and IRE1/Ask1/JNK/NOXA pathways, conclude to severe GBM lethality, highlighting new therapeutic approaches [148]. In U87 cells, 14-3-3b chaperone protein inhibits ER stress and CHOP expression and concomitant cell death, mediated by caspase activation, while simultaneously promoting β-catenin accumulation into the nucleus for pro-survival gene transcription (Figure 3) [149]. Marizomib, a BBB-permeable proteasome inhibitor, has been shown to significantly affect LN229 and U118 GBM cell lines’ viability in a time/dose-dependent manner through caspase-3, NOXA, DR5, and cytochrome upregulation and Bcl-2 downregulation. Whether UPR activation mediated the above remained unknown, but nevertheless apoptotic alterations were accompanied by BiP/P-eIF2α/CHOP and IRE1/P-JNK levels’ elevation, probably to substantiate terminal UPR activity, irrespective of oxidative and autophagic responses that did not occur in response to proteasome inhibition [150].

A number of glioma brain tumor stem cells (BTSCs) were studied to evaluate the synergistic role of salinomycin (SLM), an ER stress inducer, with TMZ in regard to UPR activation and cell death. Upon SLM treatment, higher BiP protein levels were detected, in accordance with PERK activation and XBP1 splicing. Along the same lines, an apparent autophagic flux was noticed, enhancing LC3-I lipidation towards LC3-II and autophagosomes’ formation. UPR activation led to a reduction in DNA damage repair enzymes’ expression, O6-methylguanine-DNA methyltransferase (MGMT), N-methylpurine DNA glycosylase (MPG), and DNA repair protein RAD51 homolog 1 (Rad51), associated with GBM response to TMZ (Figure 3). Thus, SLM/TMZ co-treatment resulted in severe lethality in vitro and in vivo mediated predominantly by UPR activation, implying a possible therapeutic target based on UPR regulation and consequent sensitization to the TMZ chemotherapeutic drug [151]. The concept of ER stress induction and TMZ treatment in apoptotic processes in GBM has also been expanded in distinct studies. JIK1486 is a novel ER stress inducer that synergistically with TMZ provides significant apoptotic cues for neurosphere formation and GBM growth attenuation. Cell death induced by both agents occurred due to prolonged ER stress, ATF4/CHOP pro-apoptotic function simultaneously with Rad51 downregulation and Ataxia Telangiectasia Mutated/Checkpoint kinase 2 (ATM/CHK2) activation, resulting in irreversible DNA damage and tumor doubling delay in vivo (Figure 3) [152]. Upstream of UPR signaling, overexpressed CREB3 in GBM patients and U251 cells promotes PERK/eIF2α/ATF4 activation in vitro and in vivo, leading to BAX overexpression and caspase-3 cleavage [153]. In accordance with the above, ATF4 operates as a key UPR player participating in autophagy and balance between cell survival and apoptosis. MZ-54 GBM and MEFs ATF4-KO cells undergoing ER stress induction are unable to positively regulate autophagic flux for reticulophagy and autophagosomes’ formation and maturation; thus, loss of ATF4 led to inhibition of autophagic cell death (Figure 3) [154].

The C-terminal tetrapeptide Lys-Asp-Glu-Leu receptors (KDELRs) have been characterized as oncogenic proteins, overexpressed in high-grade GBM patients, evoking tumor growth and differentiation for immune and therapy evasion. KDELR suppression not only rendered U373 GBM cells death-prone through ER stress induction, CHOP and JNK/p38 activation, but further stimulated terminal UPR and MAPK signaling upon TMZ treatment. Sensitization of GBM cells to chemotherapy was executed via BAX upregulation and in part through caspase-dependent apoptosis (Figure 3) [155]. Xanthatin, a natural anti-tumor lactone and promising product for targeting GBM progression, stimulated C6 and U251 cells’ parallel pro-apoptotic and anti-survival behavior, having caspase-3 cleaved and a high BAX/Bcl-2 ratio in a dose-dependent manner. Xanthatin induced global terminal UPR activation and apparent CHOP accumulation into cell nuclei for substantiating mitochondrial-dependent cell death in vitro and in vivo, whereas chemically induced ER stress alleviation and CHOP silencing partially rescued xanthatin-dependent apoptosis [156]. U87MG, LN229, U251, and U343 GBM cells were tested in regard to remdesivir (RDV) efficacy, a BBB-permeable anti-GBM agent, supporting its effect in GBM but not normal glial cells. U87 and U343 were the most responsive GBM cells in RDV treatment with significantly low IC50 values in vitro. Being more responsive to RDV than TMZ, the above cells in rat models displayed reduced viability in vivo, without compromising major organs’ integrity, uncontrolled weight loss, and biosafety overall. BIM upregulation and high cleaved caspase-3 positive staining followed RDV-induced upregulation of the ATF4/CHOP branch in a dose-dependent manner. As expected, blocking PERK inhibited the aforementioned events and shifted the apoptotic pathway to partial inactivation [157]. Lastly, simvastatin has been an effective agent to sensitize GBM cells to TMZ treatment by inhibiting pro-survival autophagic flux and increasing caspase-independent apoptosis. Co-treatment induced UPR activation, and even though IRE1 and eIF2α participated in autophagy inhibition, only PERK affected viability to promote cell death. Of interest was the fact that different UPR branches differentially regulated LC3-I lipidation in U87 and U251 cells, highlighting the context-dependent function of UPR in apoptosis regulation [158].

### 3.2. Multiple Myeloma

#### 3.2.1. Determining Cell Fate: The UPR Dichotomy

The importance of UPR in protein quality control in malignancies is highlighted in the regulation of B-cell differentiation and plasma cells’ transformation towards malignant MM cells. PERK expression is correlated with the abundant Ig production and secretion in mature MM cells and regulates proteostasis to oversee maintenance of cell viability [159]. UPR oncogenic role is also reflected in the elevation of bone marrow plasma cells, as well as their maturation in MM cells upon XBP1 activation. Although normal plasma cells depend on all three UPR branches to substantiate controlled Ig synthesis, dedifferentiated MM cells undergo uncontrolled transformation by altering this equilibrium in part to raise NF-kB levels excessively and allow constitutive activation, thus further accelerating XBP1 transcription rates. This loop is accompanied by IL-6 signaling, upon which MM cells in a paracrine fashion respond to IL-6 secreted by stroma cells to stimulate XBP1 activation, XBP1-dependent IL-6 expression and secretion, autophagy, and Ig production. Similarly, bone marrow stroma cells secrete TNF-α in the MM TME and provoke NF-kB cascades in MM cells. This inflammatory response readjusts TME to permit adhesion between MM and stroma cells, fibronectin-induced NF-kB activation in both cell types, and eventually disease progression [160]. The UPR mechanism also synchronizes a metabolic switch upon UPS inhibition and amino acid deprivation, sensed by ATF4. ATF4 activation favors lipid metabolism against glycolysis and induces Akt signaling to safeguard cell recovery and survival [161].

**Figure 4:**
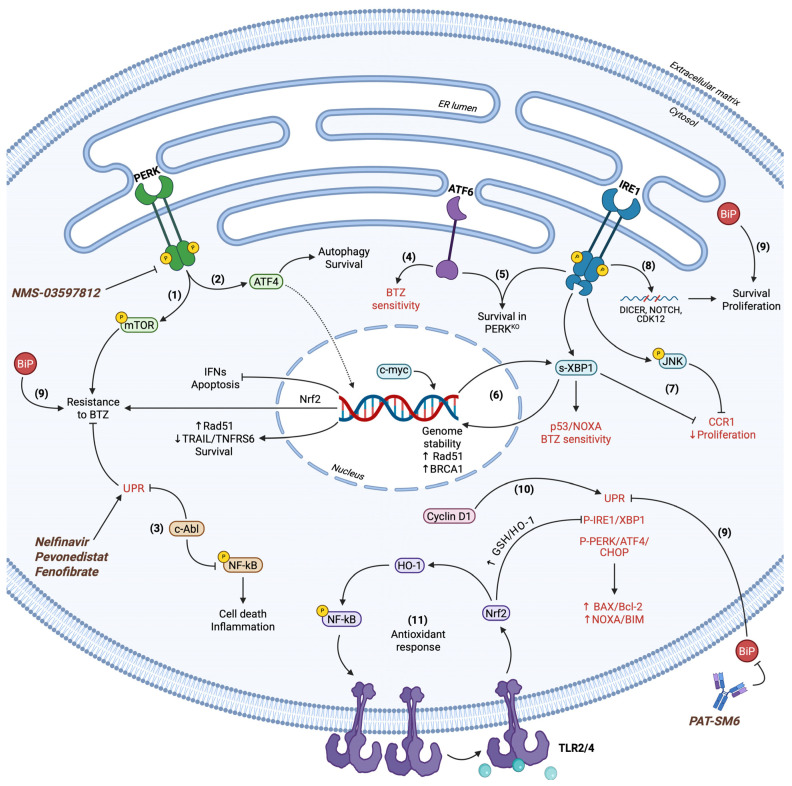
UPR in MM cell fate determination. (1) PERK regulates mTOR activity and provides resistance to BTZ. PERK inhibitors (NMS-03597812) and ER stress inducers (Nelfinavir, Pevonedistat, Fenofibrate) have been utilized in clinical trials for MM therapy. (2) ATF4 exhibits dual roles in MM survival by promoting pro-survival autophagy and the expression of key molecules, including Rad51 and Nrf-2 for genome stability and ROS neutralization, respectively. ATF4 inhibits IFNs and concomitant cell death, while simultaneously downregulates TRAIL and TNFRS6. (3) c-Abl suppresses both terminal UPR and NF-kB activity to further enhance BTZ resistance and resilience to cell death. (4) ATF6 expression correlates with BTZ sensitivity in a context-dependent manner in BTZ-susceptible MM cell lines. (5) IRE1 together with ATF6 demonstrate persistent activation to advance pro-survival cues in response to perturbed PERK signaling. (6) IRE1 and c-myc acting independently propagate s-XBP1 function to regulate gene expression in a context-dependent manner. Rad51 and BRCA1 induction safeguard cell viability, whereas s-XBP1 also regulates p53 and NOXA to substantiate apoptosis. (7) IRE1 synchronizes s-XBP1 and JNK activity to downregulate CCR1 expression and attenuate cell proliferation. (8) IRE1 via RIDD targets CDK12, DICER, and NOTCH mRNAs to promote proliferative and survival capacity. (9) BiP acts as a multifunctional cytoprotective regulator. It cooperates with UPR sensors for cell survival and tumorigenicity while simultaneously inhibiting all terminal UPR branches and downstream effectors. Hence, BAX/Bcl-2 and NOXA/Bim ratios are significantly downregulated. BiP overexpression is a marker for BTZ resistance of MM cells. Monoclonal immunoglobulin M antibody PAT-SM6 targeting extracellular BiP has been utilized in clinical trials for MM therapy. (10) Cyclin D1 provokes terminal UPR signaling in spite of its canonical roles in cell proliferation. (11) TLR2/4 signaling activates Nrf-2. Nrf-2 induces the expression of GSH and HO-1 antioxidant en-zymes. All the above independently contribute to terminal UPR inhibition and cell survival. HO-1 indirectly provokes NF-kB signaling, which in turn elevates TLR4 levels. This positive feedback loop performs independently, but in parallel with UPR signaling to abrogate the latter’s apoptotic func-tions. Terminal UPR markers are indicated in red.

The immediate need for immunoglobulin synthesis and secretion at high rates in MM cells produces an apparent protein turnover, leading to constitutively hyperactive UPR. L363, H929, U266, JJN3, RPMI-8226, OPM-2, KMS11, and JIM3 MM cell lines and CD138+ patient-derived cells pinpointed PERK protein overexpression, especially in L363, and H929 cells, which also exhibited the lowest IC50 values upon GSK2606414 treatment, a PERK kinase activity inhibitor. PERK suppression in both expression and activity entailed attenuated eIF2α/ATF4 levels in both H929 and L363 cells, having CHOP overexpressed and differential UPR activity and gene expression induction. BTZ further enhanced GSK2606414 function and highlighted that transcription regulation upon blocking PERK signaling was a pro-apoptotic and anti-survival program, executed by TNF-Related Apoptosis Inducing Ligand (TRAIL), TNFR superfamily member 6 (TNFRSF6), and Rad51 expression regulation, simultaneously with significant apoptosis-related proteins upregulation (Figure 4). Nevertheless, PERK levels do not follow a ubiquitous pattern, exhibiting differential expression in MM patient samples. Kinase activity inhibition in cells expressing high levels of PERK led to a dose-dependent loss of viability and proliferation attenuation, having lower levels of ATF4 in comparison to untreated cells. BTZ co-treatment significantly affected the above and limited CHOP expression as well. PERK inhibition followed by ER stress induction further promoted MM cells’ apoptotic behavior and aborted UPR signaling for survival attempts [162]. Bioinformatics analysis suggested that IRE1 is directly associated with MM survival and proliferation via its RIDD machinery targeting multiple mRNAs, among others cyclin-dependent kinase 12 (CDK12), DICER nuclease, and neurogenic locus notch homolog protein 1 (NOTCH1) (Figure 4) [163].

There has emerged relative evidence that depletion of either PERK, IRE1, or ATF6 UPR sensors leads to lethality of U266 and H929 cells independently of canonical mitochondrial apoptosis. PERK-KO cells surpassed impaired UPR by hyperactivating IRE1 and ATF6 pathways for maintaining a relatively healthy proteome (Figure 4). Intriguingly, MM cells did not acquire classical apoptotic morphology but inhibited mitochondrial potential, therefore apoptosome formation and apoptosis. Despite that, autophagic cell death occurred as a response, in synchronization with apoptosis blocking, forming distinct mechanisms upon which UPR operates to mediate survival through autophagy inhibition in MM cells even under mild ER stress [164]. In this setting, upon accumulation of misfolded antibodies in MM clones, BiP tends to be overexpressed and translocated towards the cell surface to mediate pro-survival or apoptotic signaling [165,166]. MM patients overexpress distinct chaperones, such as GRP94, implicated in ER homeostasis through quality control and secretion regulation downstream of UPR. Its overexpression has been associated with poor prognosis, while CD138 expression consistently correlates with GRP94 profile [167].

In SKO and RPMI MM cell lines, c-myc has been identified as a central regulator of UPR, genome stability, and survival. c-myc maintains high levels of s-XBP1 and inhibits eIF2α-induced CHOP overexpression, thus inhibiting terminal UPR. In accordance with the above, breast cancer type 1 susceptibility protein (BRCA1) and Rad51, both implicated in DNA damage repair, were found to be downregulated upon c-myc suppression, further enhancing MM apoptotic behavior. Interestingly enough, IRE1 also acts upstream of c-myc either by initiating the aforementioned c-myc function or by simulating the c-myc role through its RNase activity (Figure 4) [168]. Myeloma patients overexpress UPR markers XBP1, BiP, ATF4, and CHOP, as well as polo-like kinase 2 (PLK2). Inhibition of IRE1 kinase activity elevated the fraction of apoptotic cells upon CHOP upregulation and attenuation of XBP1 splicing. PLK2 mediated cell death due to its reduced levels upon IRE1 inhibition, while co-treatment with BTZ promoted the above phenomena (Figure 4) [169,170].

GNF-2, a c-Abl inhibitor, has shown anti-tumor effects in primary MM cells through IRE1/XBP1 activation. Distinct allosteric c-Abl inhibitor asciminib also displayed similar function by UPR, NF-kB, JAK/STAT3, and hypoxic-related upregulation to substantiate cell death. PERK signaling was central for UPR-dependent apoptosis, as shown by ATF4, ATF3, and CHOP overexpression in asciminib-treated cells, while c-Abl accumulated in the ER membrane together with calreticulin for UPR activation independent of c-Abl kinase activity (Figure 4) [171]. Distinct UPS disruptors have demonstrated differential effects in MM cells, among others RPMI-8226, RPMI-R5, MM1.s, U266, and OPM-2. H929 cells in specific features constitutively hyperactive UPR, while the pan-inhibitor of deubiquitinating enzymes, PR619, induces prodigious upregulation of IFN levels, signifying threatening conditions. BTZ paradoxically suppressed UPR markers’ expression and activation in H929 cells, implying an apoptotic state upon which UPR machinery diminishes its activity. In contrast, predominantly IRE1 and partially eIF2α-dependent translation inhibition in PR619-treated cells caused the pronounced IFN expression induction and secretion towards signal transduction in an autocrine and paracrine fashion. Eventually, IFNs precipitated an inflammatory response, acting either through the STAT1 transducer or independently of STAT, and produced active caspase-3 fragments to promote apoptosis in H929 cells (Figure 4) [172]. From an alternative perspective, studies pointed out that chemotherapy-resistant MM patient samples and cells appear to express lower levels of BiP, GRP94, Beclin-1, and LC3-II compared to sensitive samples. On the contrary, the PI3K/mTOR/Akt pathway is significantly more active in resistant samples, also having higher levels of LC3-I and lacking the prerequisite lipidation for autophagosome formation and maturation. mRNA of the above UPR markers was elevated upon ER stress induction, whereas survival markers PI3K/mTOR/Akt transcripts were decreased in U266 cells. As implied by their relative expression, ER stress conditions resulted in autophagic and UPR-dependent cell death and concurrent survival inhibition through PI3K/mTOR/Akt activity reduction [173].

#### 3.2.2. Clinical Relevance of Targeting UPR/UPS Equilibrium in Myeloma

Early findings have demonstrated in detail that BTZ-treated MM cells display dysregulated UPR and thus are predisposed to lethal ER stress and apoptosis via global terminal UPR synchronization [174]. Besides that, relevant studies in H929 and RPMI-8226 cells have illustrated that BTZ treatment increases XBP1, s-XBP1, BiP, and CHOP levels, as well as caspase activation towards cell death in a time- and dose-dependent manner. BTZ sensitivity has been widely attributed to UPR activity disturbance, and in fact XBP1-KO cells acquired apoptotic properties without being capable of utilizing UPR machinery to compensate for stress [175]. Contradictory data have perplexed the relationship between UPR/UPS function in regard to MM treatment. Even though UPR activity is essential for MM survival in a context-dependent manner, reduced ATF6 expression and ER lumen width has been associated with BTZ resistance in KMS-11 resistant cells, leading to pitfalls in MM therapy (Figure 4) [176]. Studying RPMI-8226, KMS-11, KMS-18, OPM-2, H929, and U266 cells further enhanced the notion that downregulated UPR might correlate with BTZ resilience. Accordingly, with MM cell lines and patients, in BTZ-resistant samples, low levels of XBP1 and s-XBP1 were detected, along with reduced ATF6 expression, eIF2α-induced protein synthesis inhibition, and overall attenuated immunoglobulin production. XBP1/ATF6 inactivation and concomitant chaperone expression reduction, combined with transient alleviation of ER load upon UPR relaxed activity, could possibly indicate a pathway towards BTZ acquired resistance. Induced ER and oxidative stress via TM and doxorubicin treatment, respectively, exceeded BTZ resistance, abrogated UPR fluctuations, and led to sensitization in response to UPS inhibition [177]. Different studies suggested that TM could dictate BTZ efficacy, as shown by TM-induced MM morphological alterations, differentiation, and maturation, accompanied by elevated secretion of Ig light chains and UPR-dependent transcription. Subdued XBP1 function enforces differentiation and maturity of RPMI-8226 and U266 cells, leading to restricted aggressiveness and sensitization to BTZ therapy [178]. A paradoxical trend was also noticed in RPMI-8226 and LP1 MM cells concerning cyclin D1 expression and its association with good prognosis. Cyclin D1 promoted the canonical pro-apoptotic pathway mediated by caspase activation upon ER stress induction and BTZ treatment. Similarly, cyclin D1 orchestrated imbalance in the UPR mechanism to stimulate CHOP-mediated cell death and survival inhibition (Figure 4) [179]. Additionally, MM cells exhibiting hyperactive proteasomal activity and excessive ER stress have been found to be predisposed to cell death induced by genome instability and cell cycle imbalances [180].

Twelve MM cell lines and patient-derived CD138+ cells, exhibiting heterogeneity in regard to proteasome inhibitors’ resistance, were examined for the evaluation of differential gene expression. Proteasome inhibition upon ixazomib treatment significantly altered global gene expression concerning 1553 genes, whereas resistant cells regulated only seven in vitro and ex vivo. It was noted that UPR mediated resistance via eIF2α and mTOR signaling, even though Nrf-2 was activated in sensitive cells in response to proteasome inhibition (Figure 4) [181]. BTZ-sensitive OPM-2, NCIH929, U266, and MM1.s cell lines exhibit blocked chymotrypsin-like proteasome activity and susceptibility to ER Ca^2+^ levels’ disturbance. Thus, BTZ treatment led to elevation of IRE1 activity and XBP1 splicing, simultaneously with perturbed integrated UPR function for pro-survival signaling, which in turn promoted p53/NOXA function and cell death in vitro and in vivo (Figure 4) [182]. IRE1 and proteasome inhibition with STF-083010 and ixazomib, respectively, in KMS11, RPMI-8226, and primary MM cells, and the combination of the above, have pinpointed the therapeutic aspect of UPR mechanism suppression. Loss of IRE1 function decreased viability of MM cells, whereas ixazomib further enhanced this effect, especially upon co-treatment. Intriguingly, human bone marrow mesenchymal stem cells (MSCs) rescued the viability of MM cells upon UPR and proteasome inhibition. Ixazomib mainly upregulated XBP1 mRNA to augment gene expression, counteracting loss of proteasome activity. Caspase-dependent cell death occurred due to induction of NOXA and PUMA expression, while autophagy inhibition led to apparent growth reduction and JNK-mediated apoptosis [183]. Subsequent to these propositions, the inhibition of BiP with HA15 as a single agent has been evaluated in MM progression. Although HA15 did not significantly suppress MM growth in H929 and U266 cells, BTZ and the BTZ/HA15 combination tremendously promoted apoptosis and diminished tumorigenicity in vitro and in vivo. BTZ and co-treatment provoked UPR activation, as shown by BiP, ATF4, CHOP, and XBP1 levels’ elevation, pinpointing the distinct BiP pro-survival role against global terminal UPR activation. The same expression profile was followed by BAX protein levels, probably upon UPR signaling, whereas Bcl-2 was downregulated upon BTZ and BTZ/HA15 treatment. The PERK pathway triggered pro-apoptotic mechanisms, as reaffirmed by PERK kinase activity inhibition that rescued BTZ/HA15-induced cell death (Figure 4) [184]. The complex relationship between UPR and ER stress-related chaperones in regard to BTZ resistance was highlighted in RPMI-8226, OPM-2, and MM1.s cell lines. BiP, HSP70, and other members of the HSPs were overexpressed in MM cells and further elevated upon BTZ treatment. HSP70 inhibition alone or together with BTZ treatment remarkably reduced cell viability through Bcl-2 and anti-apoptotic markers’ downregulation simultaneously with NOXA and BIM elevation. Similarly, CHOP apoptotic function was enhanced due to expression induction, leading to caspase-dependent cell death, suggesting that chaperone expression and function are required for active integrated UPR signaling, BTZ resilience, and apoptosis inhibition [185].

It is well established that Toll-like receptors (TLRs) possess a central role in MM progression. TLR4 signaling through ERK/p38 provides sufficient antioxidative response and mitochondrial protection from depolarization; therefore, TLR4 suppresses apoptotic mitophagy in UPS-compromised conditions [186]. TLR4 also counteracts BTZ efficacy in U266, H929, and OPM-2 cells through inducing heme oxygenase-1 (OH-1) expression via Nrf-2 activation. Both TLR4 and HO-1 activity inhibition prevent BTZ-dependent IRE1, PERK, BiP, and CHOP upregulation, suggesting that TLR4 utilizes UPR signaling under basal and BTZ-induced ER stress conditions to promote MM survival. In parallel, oxidative stress is relieved upon a positive feedback loop between HO-1 enzymatic activity and concomitant TLR4 upregulation, mediated by NF-kB accumulation into the nucleus that eventually accelerates TLR4-dependent pro-survival UPR (Figure 4) [187]. Apoptotic mechanisms are also regulated via TLR4 signaling to suppress terminal UPR in the presence of ER stress and BTZ. In detail, L363, H929, U266, and JJN3 cells overexpress TLR4, especially JJN3 and H929, by which PERK, P-eIF2α, ATF4, and CHOP levels are maintained relatively low. Thus, TLR4 signaling promotes proliferation and survival of MM cells and simultaneously blocks UPR-mediated cell death by differentially regulating UPR markers’ expression to mitigate the BTZ effect and provide resistance [188].

The IRE1/XBP1 branch potentiates OPM-2 and U266 cells’ sensitivity to BTZ in a context-dependent manner. It has been noted that C chemokine receptor type 1 (CCR1) expression correlates with poor prognosis in MM patients and provides BTZ-resistant properties in vitro and in vivo. This ligand-independent event occurs upon IRE1/XBP1 downregulation and concomitant JNK activity attenuation, highlighting the tumor-suppressive aspect of UPR in MM cells (Figure 4) [189]. Antithetic evidence for UPR’s role in MM pathology concerns Nrf-2 responding to heterogeneous stimuli to secure a pro-survival and proliferative cell state. Nrf-2 expression and activity appear to be enhanced upon BTZ treatment to undermine cell death induced by UPS inhibition. The aforementioned occurs partially upon direct Nrf-2-dependent CHOP downregulation to inhibit pro-apoptotic UPR. In parallel, Nrf-2 induced glutathione (GSH) expression, which in turn also downregulated CHOP indirectly, forming a positive autoregulatory loop in regard to Nrf-2 function in MM cells for depressing hostile conditions (Figure 4) [190]. Moreover, a novel MM prognostic marker, a tripartite motif family member (TRIM44), has been proposed as a potential biomarker for MM, since it has been found to be overexpressed in 858 patients with poor prognosis and resistance to BTZ. Utilizing U266 and RPMI cells, a correlational relationship between TRIM44 and UPR activation was established, in which the TRIM44 expression pattern was relatively identical with PERK/P-eIF2α/ATF4/LC3 levels. Apart from ATF4/LC3-mediated autophagy and the significant ATF4 upregulation, TRIM44 indirectly activated Nrf-2 through provoking its dissociation from Keap1. Overall, TRIM44 synchronized UPR, autophagy, and antioxidant response to alleviate BTZ-induced stress and provide pro-survival properties [191].

## 4. Therapeutic Approaches

The paramount role of ER perturbance and stress along with UPR pleiotropic activity in tumor cells have emerged as possible targets for the development and optimization of already existing therapeutic agents. Focus on the UPR system highlighted means for tumor treatment either through sensors’ inhibition or via ER stress induction, hyperactivation, and apoptosis, especially towards sensitization to chemotherapy. Along the same lines, UPR markers can serve as prognostic tools for personalized medicine and sufficient treatment. Given the binary nature of UPR in cell death and survival contradiction, there are concerns surrounding pitfalls and side effects regarding such overwhelming interventions. Nonetheless, the practice of targeting UPR in an inhibitory or activating setting has scaled up to clinical trials (Table 1), indicating a potent contribution of UPR in cancer therapy.

## 5. UPR Synergy with Extracellular Matrix in Malignancies

The cell microenvironment’s nature, consisting of cellular entities with diverse characteristics and functions, is a decisive factor in tissue homeostasis and pathophysiology. ECM, as the major non-cellular stroma component, describes the dynamic 3D scaffold upon which cells attach and form tissues, granting structural integrity and reversible cell adhesion. These properties that altogether arrange cell morphology and communication are accompanied by regulatory roles in signal transduction involved in cell growth, proliferation, migration and invasion rates, stemness capacity or differentiation, as well as programmed cell death and survival phenomena. The pool of macromolecules introduced within the ECM is comprised of secreted glycoproteins, growth factors (GFs), and cytokines with their transmembrane receptors utilizing them as ligands for signaling initiation, heterogeneous protein networks, such as collagenous fibers and laminin structures, numerous (glyco)proteins and matricellular proteins, as well as glycosaminoglycans (GAGs) and proteoglycans (PGs). CD44 is a remarkable example of a pleiotropic receptor, being characterized as a part-time PG due to its ability to carry covalently bound GAGs at its extracellular domain in a context-dependent manner. What is more, CD44 interacts non-covalently with hyaluronan (HA) GAG and with secreted serglycin (SRGN), activating numerous signaling cascades. Cellular ongoing processes are coordinated partially as a response to constant ECM remodeling through degradation and regeneration cycles, where a variety of proteases, including matrix metalloproteases (MMPs), negatively regulated by tissue inhibitors of MMPs (TIMPs), catalyze protein catabolic reactions. Apart from proteases, the plethora of secreted and transmembrane moieties eventually render ECM a crucial mediator of extracellular and intracellular interconnection [192,193].

Early signs of ECM and UPR relevance emerged from the finding that mutations in fibrillar and non-fibrillar collagen genes, as well as excessive demands for collagen biosynthesis and secretion, apply immense stress on the ER, leading to cell integrity disturbance. Recent findings have significantly elaborated on ECM/UPR interplay concerning connective tissue pathological conditions, expanding to malignant transformation and cancer progression [194]. A high-throughput study noticed a positive correlation between CD44 expression and longevity of oligodendrocyte progenitor cells (OPCs) from naked mole rats (NMR). This overexpression was accompanied by high levels of ECM and UPR-related markers. Surprisingly, CD44 function was HA independent but ATF6 dependent and provided a pro-survival signal in the presence of both CD44 and ATF6 upon ER stress. Apparently, CD44 can be found approximating ER and promote basal ATF6-induced gene expression to ensure cell viability and longevity [195]. It is also worth mentioning that plasma transmembrane components have been directly linked to ER integrity. Transmembrane protein 2 (TMEM2), which cleaves HA, has emerged as a regulatory enzyme for ER homeostasis, while CD44, which acts as an HA receptor, mediates ER stress-induced apoptosis in a context-dependent manner. MAPK pathways have also been established as signal transducers of stimuli that CD44 and UPR integrate. Understanding that ER stress can occur as a consequence of traumatic brain injury (TBI) led to the study of TBI rat models to evaluate the CD44/TMEM2 axis involvement in vivo. Results highlighted that TBI and the concomitant ER stress induction upregulate both TMEM2 and CD44, while loss of TMEM2 concludes to CD44 reduced levels. TBI models also experienced ERK/p38-induced cell death, further deteriorating upon TMEM2 knockdown. Thus, it was concluded that TBI pathology was offset via TMEM2/CD44 through ERK/p38 downregulation in a time-dependent manner to block caspase-dependent apoptosis and provide an adaptation advantage [196]. The same mediators appear to influence mammalian cells’ longevity, survival, and resistance to ER stress in different ways in different settings. TMEM2 function resulting in HA degradation to low-molecular-weight HA (LMW-HA) can activate CD44 through direct binding, which in turn promotes MAPK (ERK/p38) signaling independently of UPR. Simultaneously, UPR activation, based on IRE1/JNK and IRE1 RNase activity, can regulate cell fate in both ways, depending on ER stress intensity. Nevertheless, MAPK responding to the TMEM2/LMW-HA/CD44-dependent cascade can maintain cells viable, exhibiting exclusively pro-survival action and counteracting the IRE1-induced terminal UPR upon ER stress [197,198].

**Figure 5:**
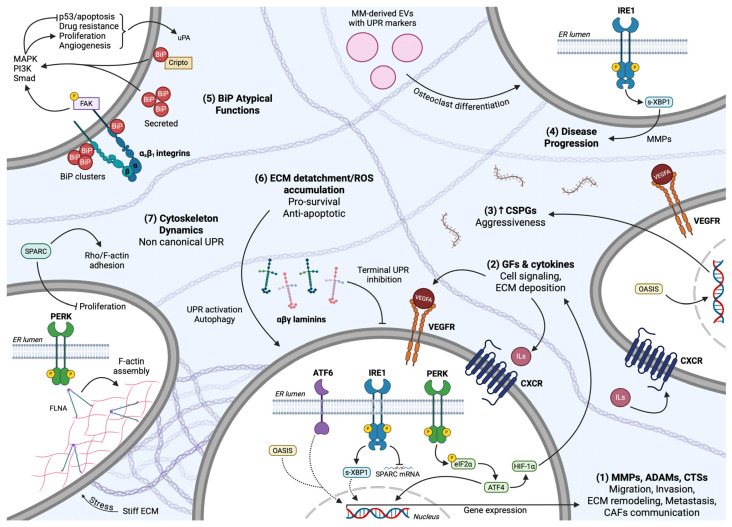
Communication between ECM and UPR. (1) All URP branches induce the expression of extracellular proteases to catalyze ECM remodeling and concomitant migration, invasion, metastasis, and interplay with surrounding CAFs. (2) Canonical UPR signaling and ATF4-dependent HIF-1 transactivation upregulate GF and cytokine expression and secretion to substantiate ECM deposition and cell signaling in an autocrine and paracrine fashion. (3) OASIS activity correlates with CSPG deposition and GBM aggressive phenotype. (4) MM-derived EVs containing UPR markers are encapsulated by osteoclasts, stimulating IRE1 activity, MMPs expression, and MM-related bone disease progression. (5) Cell-surface BiP cooperates with αxβ1 integrin/FAK axis and interacts with Cripto extracellular protein to trigger MAPK, PI3K/Akt, and Smad signaling, while secreted BiP regulates the survival/cell death equilibrium to favor BTZ resistance of MM cells and apoptosis inhibition. (6) Compromised ECM nature upon cell detachment resulting in ROS accumulation is counteracted through UPR activity, together with pro-survival autophagic flux. Laminins contribute to ECM fidelity and cell homeostasis by suppressing terminal UPR. (7) Stiff ECM is monitored as a stress-inducing condition and commands for PERK overexpression, allowing PERK/FLNA interaction and F-actin assembly to readjust cytoskeleton dynamics and adaptation of GBM cells in their TME. IRE1 cleaves and depletes SPARC mRNA, leading to excessive proliferation rate, altered Rho/F-actin architecture, and attenuated migration.

Given that ECM illustrates a central role in cancer progression through its multifunctionality, tumorigenic events have been studied to evaluate the aspect of ER stress in correlation with ECM function, as well as its synergy with the UPR pathway. Triple negative breast cancer (TNBC) cells tend to display hyperactive PERK signaling in cases of distant metastasis, while ATF4 has been shown to induce MMP-2 and MMP-7 expression to promote ECM remodeling, therefore invasion and metastasis of esophageal squamous cell carcinoma and lymph node in vitro and in vivo [199,200]. Likewise, high levels of s-XBP1 are responsible for the overexpression of MMP-1, MMP-3, and MMP-9 in oral and esophageal squamous cell carcinomas, respectively, while both ATF4 and ATF6 induce a disintegrin and metalloprotease 7 (ADAM7) expression and secretion in breast, ovarian, and prostate cancer [201,202,203]. ECM structure alterations that depend on UPR activity have been pinpointed to affect other malignant tumors, among others chronic myeloid leukemia cells that maintain phosphorylated eIF2α status constitutively elevated to induce the expression of MMPs and cathepsins, being afterwards recruited within the TME to substantiate ECM remodeling and communication between tumor cells and cancer-associated fibroblasts (CAFs) (Figure 5) [204]. In GBM cells, UPR aims are not limited to MMPs’ expression and secretion but also include GFs, chemokines, and inflammatory mediators, such as vascular endothelial growth factor A (VEGFA), ILs, and specifically IL-1β, IL-6, and IL-8, synchronizing an IRE1-induced attempt to augment pro-tumorigenic signaling (Figure 5) [205,206,207].

The PERK branch has been linked with autophagy and redox homeostasis in mammary epithelial cells (MECs) and breast cancer pathology upon cell detachment from their ECM. As such, the ECM hostile microenvironment produces an abundance of ROS and advances to an apoptotic phenotype of breast cancer cells. This outcome is mitigated by the PERK/eIF2α/ATF4/CHOP pathway that promotes LC3-I to LC3-II conversion, autophagy-related proteins’ (ATGs) expression, and facilitates autophagosomes’ formation. This ECM-induced intracellular recycling leads to UPR-mediated pro-survival efforts for adaptation and concurrently neutralizes ROS excess (Figure 5) [208]. Tumor extracellular vesicles (TEVs) and their role in biomolecules’ trafficking within the ECM towards target cells have emerged as a key factor in cancer progression. Interestingly, TEVs isolated from the high-grade human urothelial carcinoma TCCSUP cell line and exposure of nonmalignant human SV-HUC urothelial cells to these TEVs have shown a tremendous alteration in the latter’s properties. The SV-HUC cell’s cycle was readjusted upon TEV treatment and led to uncontrolled proliferation, resistant to cell–cell contact inhibition both in vitro and in vivo. What is more, N-cadherin and E-cadherin levels were found to be upregulated and downregulated, respectively, reaffirming the invasive capacity and morphological alterations that TEVs provoked. This malignant transformation was accompanied by genome instability and ROS generation that SV-HUC negated through encoding antioxidant enzymes and activating UPR. Although TEV-induced ER stress was initially damaging for SV-HUC that activated the apoptotic PERK/ATF4/CHOP pathway, prolonged exposure led to IRE1 activation, ER stress alleviation, and apoptosis inhibition, with PERK signaling being intriguingly attenuated. Eventually, IRE1 acting upstream of NF-kB to initiate a pro-survival inflammatory response safe-guarded SV-HUC cell’s fidelity and remodeled ER/mitochondria organelles to counterpoise proteotoxicity and unresolved stress [209]. Given the formidable role of MM cells in TME reorganization and cell communication, RAW264.7 cells, an in vitro model of osteoclast formation, were treated with MM1.s and U266-derived EVs. Proteomic analysis revealed that MM EVs contained UPR-related markers, and the IRE1/XBP1 pathway was activated in RAW264.7 cells upon EVs treatment. As a consequence, nuclear factor of activated T-cells, cytoplasmic 1 (NFATc1) transcription was induced for terminal osteoclast differentiation and MMP-9 expression, which were dependent on both MM and osteoclasts. IRE/XBP1 function for adequate bone resorption, signifying IRE1 as a possible target in MM and TME osteoclasts for eliminating myeloma bone disease (Figure 5) [210]. Among others, laminin glycoproteins, forming heterotrimeric (αβγ) complexes and networks, have been shown to affect UPR status in a context-dependent manner. Laminin α5 knockdown in the HT-19 colorectal adenocarcinoma cell line overall attenuated their pro-tumorigenic properties and provoked an ER stress-induced response. More specifically, IRE1a and XPB1, simultaneously with their and PERK target genes, were found upregulated upon laminin α5 suppression, as well as mTORC1 inhibitors, signifying a less pluripotent phenotype prone to cell death, partially mediated by terminal UPR signaling (Figure 5) [211].

UPR activation in response to ECM readjustment and vice versa was also noticed in studies concerning neuroblastoma’s cytoprotective mechanisms in compromised TMEs. NB1691 and SK-N-AS neuroblastoma cell lines express and secrete excessive VEGFA upon both ER stress and hypoxic conditions, due to UPR and hypoxia-inducible factor (HIF) activation, respectively. Predominantly ATF4 and, to a lesser extent, XBP1 bind to the promoter of VEGFA and promote its expression, while HIF-1α displays similar functions. Simultaneous induction of ER stress and hypoxia led to a greater effect, but surprisingly this did not occur as a cooperative attempt; rather, UPR transactivated HIF-1α to further enhance VEGFA expression and ECM deposition, independently of ATF4 activity (Figure 5) [212]. Distinct TFs simulating UPR function have also been associated with ER relevance to ECM. Old astrocyte specifically induced substance (OASIS) belongs to these TFs that resemble ATF6 in regard to localization, structure, activation, and gene expression induction. U373, A172, and U87 GBM cell lines, expressing high levels of OASIS, have been tested for their response to ER stress upon OASIS knockdown, and not only was the expression of BiP and GRP94 chaperones reduced, but chondroitin sulfate PGs (CSPGs) levels were also diminished. This was accompanied by phenotype alterations and migration rate attenuation but did not significantly affect either survival or proliferation capacity, underlying the role of UPR-related markers in the aggressiveness of tumor cells rather than cell death resistance via TME remodeling (Figure 5) [213].

Recent insights into glioma pathology have established IRE1 as a key player in ECM remodeling and cell behavior. IRE1 significantly affects U87 cell’s cytoskeleton organization in vitro and in vivo and exhibits a dual role by promoting proliferation while restricting Rho/F-actin-mediated focal adhesions’ formation and infiltration in surrounding structures, highlighting a potential dissociation between GBM tumor growth and migration, regulated by IRE1. This IRE1 effect was found to be XPB1-dependent and RIDD-dependent simultaneously. Specifically, the phenotype IRE1-knockout U87 cells acquired was probably a consequence of XBP1 cleavage attenuation and its TF activity loss, which led to collagen types, fibrillin 1, fibronectin, and MMP-2 upregulation, as well as TIMP-1 downregulation, providing a possible explanation of their enhanced invasiveness and migratory capability. Secreted protein acidic and rich in cysteine (SPARC), an ECM matricellular protein also known as osteonectin, was found in the center of IRE1-induced phenomena, since IRE1 targets and cleaves SPARC mRNA via its RNase activity. This downregulation in turn influences ECM deposition, cell–matrix interactions, and inhibitory signals in cell cycle progression. In fact, SPARC functions in an autocrine/paracrine mechanism to inhibit proliferation and tumor formation of glioma cells and enhance FAK/Rho/F-actin-mediated migration and invasion, whereas IRE1 hydrolyses SPARC mRNA to induce the opposite effect (Figure 5) [214]. Intriguingly, TME can simultaneously guide UPR function independent of its role as a signal transduction machinery. ECM dynamic network undergoes consistent remodeling that could correspond to stiff GBM TME. Oncogenic stiffness and mechanical stress are monitored by F-actin cytoskeleton filaments that reorganize tumor cells into an elongated and invasive state. As shown in detail, PERK mediates this response by overexpressing filamin A (FLNA) and F-actin while interacting simultaneously with FLNA, allowing it to assemble F-actin networks. This PERK-driven event is necessary for responding to mechanical stimuli that eventually promote F-actin polymerization, adaptation of GBM cells into stiff microenvironments, and their aggressive properties in regard to proliferation and migration rates. Notably, this positive feedback loop is further enhanced by PERK upregulation in a stiffness-dependent manner, whereas ATF4 and the canonical PERK downstream pathway is not required for this adaptive effort (Figure 5) [215]. Through utilizing GG16-WT and GG16-PERK-KO GSC cells, it was concluded that PERK acts as a central regulator of adaptation upon stiffness induction and extreme mechanotransductional alterations. More specifically, PERK-dependent F-actin polymerization allowed GG16-WT cells to undergo differentiation and reorganize essential cytoskeleton structures, such as vinculin, talin, and vimentin, promoting their expression and assembly, especially due to stiffness-induced stimuli. Thus, expression of PERK was found to be correlated with an advantageous response to stiff TME, upon which GSCs acquired significantly elongated morphology. As mentioned above, F-actin and FLNA recruitment, simultaneously with glial filament acidic protein (GFAP) upregulation in a stiffness-dependent manner, indicating GSCs’ differentiation, were not dependent on PERK kinase activity and signaling but on the initial PERK/FLNA interaction. Loss of PERK disallowed F-actin polymerization and, interestingly enough, induced an apparent overexpression of tubulin that exhibited an opposite expression pattern against F-actin, suggesting that PERK possesses a novel, kinase-independent property as an elegant switch between cytoskeleton dynamics in response to extracellular mechanical signals (Figure 5) [216].

Of interest was the assessment of BiP atypical function in tumors that correlates with its spatial localization intracellularly and extracellularly. BiP has been found in the cell surface of tumor cells, among other lymphocytes, neuroblastoma, lung, colon adenocarcinoma, ovarian, melanoma, osteosarcoma, gastric, pancreatic, and breast cancer cells. Cell-surface BiP, acting as a co-receptor, can bind to different regulatory membrane proteins implicated in cell signaling and cancer progression [217]. Cripto, an extracellular glycosylphosphatidylinositol membrane-anchored oncogenic protein, interacts with BiP, regulating transforming growth factor β (TGFβ)/Smad and ERK/Akt pathways to promote malignant phenotypes in human epithelial cancer cells (Figure 5) [218]. In pancreatic ductal adenocarcinoma cancer stem-like cells (CSCs), panc-1 and gemcitabine recruited all three UPR branches to orchestrate a global pro-survival mechanism and inhibit pro-apoptotic pathways. On the contrary, gemcitabine-induced extreme ER stress conditions gave rise to caspase-dependent terminal UPR. UPR exploited ATF6f to substantiate the pro-survival attempt through upregulating BiP and inducing the β-catenin pathway in a BiP-dependent mechanism not yet fully understood. This ATF6/BiP/β-catenin cascade led to the overexpression of the urokinase plasminogen activator (uPA) at both transcription and translation levels. Extracellular protease uPA is implicated in ECM remodeling and epithelial–mesenchymal transition (EMT); therefore, its upregulation resulted in the enhancement of panc-1 aggressive properties. Besides that, uPA was shown to regulate p53 as a response to gemcitabine treatment to augment panc-1 survival by perturbing mitochondrial-induced apoptosis phenomena (Figure 5) [219]. uPA has also been pinpointed as the terminal BiP target in different scenarios. Studies on HT29, SW480, SW620, DLD1, and Lovo colorectal cancer cell lines showed an unexpected distribution of BiP throughout the cell membrane, forming homogenous clusters. These BiP clusters not only co-localize with focal adhesion kinases (FAK) but also simultaneously interact with β1 integrins to regulate cell motility and mediate outside-in integrin signaling in parallel with FAK. BiP complexes facilitate their matrix-dependent function by further enhancing ECM remodeling and, more specifically, by being in close proximity with the uPA/uPA receptor (uPAR) system to activate MMPs and reorganize ECM structure for cancer cells’ invasive and migratory capacity (Figure 5) [220]. To understand the cell-surface BiP role in TME dynamics in cancer-related events, human umbilical vein endothelial cells (HUVECs) have been used for tumor neovascularization studies. HUVECs displayed an apparent accumulation of BiP on their cell membranes, which was significantly elevated upon VEGF treatment. VEGF-treated HUVECs utilized BiP as an important mediator for ERK, phospholipase C (PLC), and VEGF receptor 2 (VEGFR2) activation to upregulate their proliferation rate and stimulate angiogenesis [221]. Expanding on the concept of BiP/ECM cooperation in vascularization, while having established that BiP can be found secreted within the ECM, solid tumor and myeloma cell lines were utilized as models for evaluating the effect of BiP on BTZ resistance. BTZ-resistant tumor cells’ microenvironment was found abundant with secreted BiP that counteracted BTZ function, independently of proteasome activity. Although the OPM-2 myeloma cell line did not secrete high levels of BiP, the introduction of recombinant BiP in the OPM-2 medium rescued the bortezomib-induced effect. Interestingly enough, these findings suggest that BiP induced pro-survival ERK and Akt signaling, whereas p53-dependent apoptosis occurred only due to BiP depletion, providing new insights into the cell’s response to hostile conditions under the guide of a complex crosstalk and non-canonical signaling (Figure 5) [222].

## 6. Conclusions

Cell physiology is directly regulated by the UPR pathways, functioning as intrinsic homeostatic mechanisms to safeguard cell viability. The wide array of phenomena affected by proteostasis imbalances, ER stress, and UPR activity have revealed the paramount role of UPR in cell signaling and communication, expanding from acute pathologies to oncogenesis and tumor progression. UPR can also integrate signals derived from extracellular stimuli and simultaneously initiate or mediate intracellular alterations to determine cell properties and cell fate. Additionally, tumorigenicity and drug resistance have been attributed at least partly to persistent and excessive UPR activation, even in the absence of upstream signaling cascades. PERK, IRE1, and ATF6 sensors and downstream effectors, acting independently or cooperatively, can exhibit dual effects on the aggressiveness of malignant cells. In parallel, intracellular, cell-surface, and secreted BiP chaperones provoke non-canonical UPR signaling and directly contribute to tumor growth and survival, pinpointing the UPR paradoxical mechanism of action in a context-dependent manner. Nevertheless, the continuing effort for drug discovery has led to a targeted approach respecting ER stress induction and UPR manipulation in cases of multiple myeloma, glioblastoma, and other malignancies [223,224].

## Figures and Tables

**Table 1:** Ongoing clinical trials for the development of UPR-targeted therapies.

Agent(s)—Combined Treatment	Tumor	Phase (ID)
Nelfinavir i. UPR activation ii. Sensitization to BTZ	Multiple myeloma	II (NCT02188537)
Nelfinavir and lenalidomide/ dexamethasone i. UPR activation ii. Sensitization to BTZ	Multiple myeloma	I/II (NCT01555281)
1. Ixazomib (MLN9708) i. Proteasome inhibition 2. Fulvestrant i. Estrogen receptor-downregulating antiestrogen ii. Estrogen receptor block UPR induction	ER-positive breast cancer	Ib (NCT02384746)
1. Pevonedistat i. ER stress/terminal UPR induction 2. Vincristine, dexamethasone, PEG-asparaginase, and doxorubicin (VXLD) i. Overall cytotoxicity	Acute lymphoblastic leukemia and Lymphoblastic non-Hodgkin lymphoma	I (NCT03349281)
Monoclonal immunoglobulin M antibody PAT-SM6 i. BiP recognition/inhibition	Multiple myeloma	I (NCT01727778)
Fenofibrate i. Terminal UPR activation	Multiple myeloma	II (NCT01965834)
1. Minnelide i. ER stress induction 2. Abraxane and gemcitabine i. Standard of care drugs	Metastatic adenocarcinoma of the pancreas	Ib (NCT05557851)
1. Botensilimab (AGEN1811) and Balstilimab (AGEN2034) i. Immune-based therapy for ER stress/UPR induction 2. Nab-paclitaxel, gemcitabine and cisplatin i. Triple chemotherapy 3. Chloroquine i. Autophagy inhibition 4. Celecoxib i. Inflammation inhibition	Metastatic pancreatic cancer	I (NCT06076837)
NMS-03597812 i. PERK inhibition	Multiple myeloma	I (NCT05027594)
BTZ i. UPS inhibition	Recurrent glioma	I (NCT00006773)
1. Marizomib i. UPS inhibition (BBB permeable) 2. TMZ i. Alkylating agent 3. Radiotherapy	Brain cancer	I (NCT02903069)
1. Marizomib i. Alkylating agent 2. Bevacizumab i. Angiogenesis inhibition	Glioblastoma grade IV	I/II (NCT02330562)

## Data Availability

No new data were created or analyzed in this study.

## Abbreviations

ADAM7	A disintegrin and metalloprotease 7
ARE	Antioxidant response element
ASK1	Apoptosis-signal-regulating kinase 1
ATF4	Activating transcription factor 4
ATF6	Activating transcription factor 6
ATF6f	ATF6 fragment
ATGs	Autophagy-related proteins
ATM/CHK2	Ataxia Telangiectasia Mutated/Checkpoint kinase 2
BAD	Bcl-2 antagonist of death
BAK	Bcl-2 antagonist/killer protein
BAX	Bcl-2-associated X protein
BBB	Blood-brain barrier
Bcl-2	B-cell leukemia/lymphoma 2 protein
BIM	Bcl-2 interacting mediator of cell death
BiP	Binding immunoglobulin protein
Blos1	Biogenesis of lysosome-related organelles 1 subunit 1
BRCA1	Breast cancer type 1 susceptibility protein
BTSCs	Brain tumor stem cells
BTZ	Bortezomib
b-ZIP	Basic leucine zipper
CAFs	Cancer-associated fibroblasts
CCR1	C chemokine receptor type 1
CDK12	Cyclin-dependent kinase 12
CHOP	C/EBP homologous protein
COPII	Coat protein complex II
CREB	cAMP-response element binding protein
CREBH	CREB hepatocyte
CSCs	Cancer stem-like cells
CSPGs	Chondroitin sulfate proteoglycans
DR5	Death receptor 5
DTT	Dithiothreitol
DUBs	Deubiquitinases
2-DG	2- deoxy-D-glucose
ECM	Extracellular matrix
EGF	Epidermal growth factor
EGFR	Epidermal growth factor receptor
eIF2α	Eukaryotic translation initiation factor 2α
EMT	Epithelial-mesenchymal transition
ER	Endoplasmic reticulum
ERAD	ER-associated degradation
ERK	Extracellular signal-regulated kinases
FAK	Focal adhesion kinases
FLNA	Filamin A
FOXF1	Forkhead box transcription factor 1
5-FU	5-Fluorouracil
GADD34	DNA damage-inducible 34
GAGs	Glycosaminoglycans
GBM	Glioblastoma multiforme
GF	Growth factor
GFAP	Glial filament acidic protein
GPT	N-acetylglucosamine-1-phosphate transferase
GSCs	Glioma stem cells
GSH	Glutathione
HA	Hyaluronan
HDR1	Hydroxymethyllutaryl reductase degradation protein 1
HIF	Hypoxia-inducible factor
HK2	Hexokinase 2
HO-1	Heme oxygenase-1
HSP70	Heat shock protein 70
HUVECs	Human umbilical vein endothelial cells
IFN-γ	Interferon-γ
IKK	Inhibitor of nuclear factor-kB kinase
ILs	Interleukins
IRE1	Inositol requiring enzyme 1
JNK	c-Jun N-terminal kinase
KDELRs	Lys-Asp-Glu-Leu receptors
KPNB1	Nuclear import receptor karyopherin β1
LC3	Microtubule-associated protein 1A/1B-light chain 3
LMW-HA	Low-molecular-weight hyaluronan
MAPK	Mitogen-activated protein kinases
MECs	Mammary epithelial cells
MEFs	Mouse embryonic fibroblasts
MEK	Mitogen-activated protein
MGMT	O6-methylguanine-DNA methyltransferase
MM	Multiple myeloma
MMPs	Matrix metalloproteases
MPG	N-methylpurine DNA glycosylase
MSCs	Mesenchymal stem cells
mTOR	Mammalian target of rapamycin
NFATc1	Nuclear factor of activated T-cells cytoplasmic 1
NF-kB	Nuclear factor-kB
NK	Natural killer
NMR	Naked mole rats
NOTCH1	Neurogenic locus notch homolog protein 1
NOXA	Phorbol-12-myristate-13-acetate-induced protein 1
NOX2/4	NADPH oxidases 2/4
Nrf-2	Nuclear factor erythroid 2-related factor 2
OASIS	Old astrocyte specifically induced substance
OPCs	Oligodendrocyte progenitor cells
PERK	Double-stranded RNA-activated protein kinase (PKR)–like ER kinase
PGs	Proteoglycans
PLC	Phospholipase C
PLK2	Polo-like kinase 2
PUMA	p53-upregulated modulator of apoptosis
Rad51	DNA repair protein RAD51 homolog 1
RCAN1	Regulator of calcineurin 1
RDV	Remdesivir
RER	Rough ER
RIDD	IRE1-dependent mRNA decay
RIP	Regulated intermembrane proteolysis
RNase	Ribonuclease
ROS	Reactive oxygen species
SER	Smooth ER
SLM	Salinomycin
SPARC	Secreted protein acidic and rich in cysteine
SPHK1/2	Sphingosine kinase 1/2
SRGN	Serglycin
S1P/S2P	Site-1 protease/Site-2 protease
TBI	Traumatic brain injury
TEVs	Tumor extracellular vesicles
TFs	Transcription factors
TGFβ	Transforming growth factor
Tg	Thapsigargin
TIMPs	Tissue inhibitors of matrix metalloproteases
TIS	Therapy-induced senescence
TLRs	Toll-like receptors
TM	Tunicamycin
TME	Tumor microenvironment
TMEM2	Transmembrane protein 2
TMZ	Temozolomide
TNBC	Triple negative breast cancer
TNF	Tumor necrosis factor
TNFR1	Tumor necrosis factor receptor 1
TNFRSF6	Tumor necrosis factor receptor superfamily 6
TRAF2	Tumor necrosis factor receptor-associated factor 2
TRAIL	Tumor necrosis factor-Related Apoptosis Inducing Ligand
TRIB3	Tribbles homolog 3
TRIM44	Tripartite motif family member
UBL5	Ubiquitin-like protein 5
uPA	Urokinase plasminogen activator
uPAR	Urokinase plasminogen activator receptor
UPR	Unfolded protein response
UPS	Ubiquitin-proteasome system
UPS19	Ubiquitin specific protease 19
VEGFA	Vascular endothelial growth factor A
VEGFR2	Vascular endothelial growth factor receptor 2
WARS1	Tryptophanyl-tRNA synthetase 1
XBP1	X-box binding protein 1
s-XBP1	Spliced-XBP1
u-XBP1	Unspliced-XBP1
XPOT	Exportin-T
